# Influence of Mutations and N-Glycosylation Sites in the Receptor-Binding Domain (RBD) and the Membrane Protein of SARS-CoV-2 Variants of Concern on Antibody Binding in ELISA

**DOI:** 10.3390/biology13040207

**Published:** 2024-03-23

**Authors:** Mandy Schwarze, Daniela Volke, Juan Camilo Rojas Echeverri, Robin Schick, Nicole Lakowa, Thomas Grünewald, Johannes Wolf, Stephan Borte, Markus Scholz, Andor Krizsan, Ralf Hoffmann

**Affiliations:** 1 Institute of Bioanalytical Chemistry, Faculty of Chemistry and Mineralogy, Universität Leipzig, 04103 Leipzig, Germany; mandy.schwarze@uni-leipzig.de (M.S.);; 2 Center for Biotechnology and Biomedicine, Universität Leipzig, 04103 Leipzig, Germany; 3 Klinik für Infektions- und Tropenmedizin, Klinikum Chemnitz gGmbH, 09113 Chemnitz, Germanyt.gruenewald@skc.de (T.G.); 4 Department of Laboratory Medicine, Hospital St. Georg gGmbH, 04129 Leipzig, Germany; 5 Immuno Deficiency Center Leipzig, Jeffrey Modell Diagnostic and Research Center for Primary Immunodeficiency Diseases, Hospital St. Georg gGmbH, 04129 Leipzig, Germany; 6 Institute for Medical Informatics, Statistics and Epidemiology, Universität Leipzig, 04107 Leipzig, Germany; 7 LIFE Research Center of Civilization Diseases, Universität Leipzig, 04103 Leipzig, Germany

**Keywords:** ELISA, epitope, immunoglobulins G (IgG), glycan mapping, N-glycosylation, SARS-CoV-2, RBD

## Abstract

**Simple Summary:**

Many proteins are glycosylated, for example, on asparagine residues, which can affect protein function in a variety of ways, but can also shield immunogenic sites in viral proteins from the immune system. This is also the case for the highly glycosylated SARS-CoV-2 proteins. In this study, different receptor-binding domains (RBDs) of several SARS-CoV-2 variants of concern (VOCs), a subunit of the highly glycosylated spike protein, and the membrane protein, which has not been extensively studied, were produced in human cells and bacteria for the relevant VOCs. The resulting RBDs were analyzed for their N-glycosylation sites by mass spectrometry and for their ability to bind to IgG antibodies produced in response to SARS-CoV-2 infection by ELISA.

**Abstract:**

Severe acute respiratory syndrome coronavirus 2 (SARS-CoV-2) can infect human cells by first attaching to the ACE-2 receptor via its receptor-binding domain (RBD) in the spike protein. Here, we report the influence of N-glycosylation sites of the RBD and the membrane (M) protein on IgG antibody binding in serum samples from patients infected with the original SARS-CoV-2 strain in Germany. The RBDs of the wildtype, alpha, beta, gamma, and kappa variants expressed in HEK293S GnTI− cells were all N-glycosylated at Asn331, Asn334, Asn343, and Asn360 or Asn370, whereas the M-protein was glycosylated at Asn5. An ELISA using a coated RBD and probed with anti-RBD IgG antibodies gave a sensitivity of 96.3% and a specificity of 100% for the wildtype RBD, while the sensitivity decreased by 5% to 10% for the variants of concern, essentially in the order of appearance. Deglycosylation of the wildtype RBD strongly reduced antibody recognition by ~20%, considering the mean of the absorbances recorded for the ELISA. This effect was even stronger for the unglycosylated RBD expressed in *Escherichia coli*, suggesting structural changes affecting epitope recognition. Interestingly, the N-glycosylated M-protein expressed in HEK293S GnTI− cells gave good sensitivity (95%), which also decreased to 65% after deglycosylation, and selectivity (100%). In conclusion, N-glycosylation of the M-protein, the RBD, and most likely the spike protein are important for proper antibody binding and immunological assays, whereas the type of N-glycosylation is less relevant.

## 1. Introduction

Severe acute respiratory syndrome coronavirus 2 (SARS-CoV-2) is a single-stranded RNA virus that belongs to the genus betacoronavirus, similar to the previously emerging SARS and Middle East Respiratory Syndrome (MERS) coronaviruses. It is the seventh coronavirus that can spread among humans and the associated coronavirus disease 2019 (COVID-19) leads to mild (fever, cough, headache, diarrhea) to severe symptoms, such as respiratory failure and other long-term symptoms [1,2]. The virus particles consist of four structural proteins: spike (S), envelope (E), membrane (M), and nucleocapsid proteins (N), which are important for host recognition, binding, recycling, and pathogenesis of the virus [3,4]. The M-protein organizes the assembly and structure of new virions by forming a mushroom-shaped dimer, which then assembles into higher order oligomers [5,6]. The S-protein contains the receptor-binding domain (RBD), which plays a key role in recognizing human cells by binding to the human ACE-2 receptor, allowing the virus to enter and ultimately infect cells [7,8].

Due to the strong and rapid spread of the virus and the lack of therapeutic interventions at the beginning of the COVID-19 pandemic, a variety of potential therapeutic approaches were proposed and tested, but vaccination was the first effective method to overcome the pandemic by effectively preventing SARS-CoV-2 infections. The first vaccines were marketed in late 2020, particularly mRNA-based vaccines that induce an immune response against the S-protein [9,10,11]. The S-protein is a glycoprotein that has a total of 22 N-glycosylation and 17 potential O-glycosylation sites, including two N- and two O-glycosylation sites, within the RBD [12]. These glycosylation sites shield immunogenic regions from host antibodies, preventing a strong immune response and weakening antibody binding, a common mechanism used by viruses to evade the immune response. Considering the importance of N-glycosylation, Huang et al. explored another therapeutic approach, using an N-glycosylation inhibitor targeting the glycosyltransferase STT3A, which resulted in non-glycosylated S-proteins with enhanced binding affinity for neutralizing antibodies, thereby reducing viral infectivity [13].

N-glycosylation occurs in the endoplasmic reticulum (ER), where the glycan precursor, consisting of two N-acetylglucosamine (GlcNAc) units, three glucose (Glc) units, and nine mannose (Man) units, is attached to specific asparagine residues [4,14,15]. In recombinant S-proteins, the N-glycan structure depends on the cell line used for protein expression. HEK293 cells produce S-proteins with more complex glycans, while S-proteins produced in Vero E6 cells show a significantly higher proportion of high-mannose glycans [16]. The RBD is typically glycosylated in these cell lines at Asn331 and Asn343, while an additional glycosylation site at Asn334 was reported for insect cells [4]. Interestingly, the entry of pseudotyped viruses was dramatically reduced in studies on S-proteins mutated at Asn331 and Asn343, abolishing glycosylation at these sites and indicating their importance for viral infection [17]. Thus, the expression system has a strong influence on the glycosylation sites, which will also affect the properties of the expressed S-protein. Studies of the glycosylation pattern of S-proteins expressed in HEK293 cells showed that the N-glycosylation pattern was identical for all sequences of SARS-CoV-2 variants of concern (VOC), from the original Wuhan strain to Omicron BA.1 [12,18,19]. The M-protein has been much less studied, with experimental confirmation of the predicted N-glycosylation sites still missing [4,12].

The current study focused mainly on the RBD of the S-protein expressed in HEK293S cells, considering the mutations in the RBD of the most relevant VOCs observed in Europe: RBD constructs corresponding to the wildtype, alpha (B.1.1.7, N501Y), beta (B.1.351, K417N, E484K, and N501Y), gamma (B.1.1.28.1, K417T, E484K, and N501Y), kappa (formerly delta, B.1.617.1, L452R, and E484Q), and omicron BA.1 (G339D, S371L, S373P, S375F, K417N, N440K, G446S, S477N, T478K, E484A, Q493K, G496S, Q498R, N501Y, and Y505H). These RBDs and the M-protein expressed in HEK293S cells were screened for N-glycosylation at the predicted Asn residues and used in ELISA, a well-established and routinely used assay to test antibody titers present in serum samples and to map epitopes of antibodies [20,21]. Based on previous results suggesting that N-glycosylation prevents or reduces antibody binding [22], the influence of N-glycosylation on IgG antibody recognition of the RBD and M-protein was studied using ELISA. The aim of this study was to test sera from COVID-19 patients against glycosylated, deglycosylated, and unglycosylated RBDs and M-proteins to assess the relevance of protein N-glycosylation for antibody recognition and to test whether bacterial expression systems would be sufficient for RBD and M-protein expression.

## 2. Materials and Methods

Reagents were obtained from the following companies: Advansta Corporation (San Jose, CA, USA): WesternBright Sirius^®^; AppliChem GmbH (Darmstadt, Germany): Iodoacetamide (IAA); Biosolve BV (Valkenswaard, The Netherlands): Dimethylformamide (DMF, peptide synthesis grade) and piperidine (≥99.5%); Carl Roth GmbH & Co. KG (Karlsruhe, Germany): Carbenicillin disodium salt, Dithiothreitol (DTT), lysogeny-broth (LB) medium, lysozyme (≥45,000 FIP U/mg), ROTI^®^Stock 10× PBS, ROTI^®^Stock 10× PBS-T, sodium chloride (≥99.5%), sodium dodecyl sulfate (SDS, ≥99.5%), sulfuric acid, terrific-broth (TB) medium, thioanisole (≥99%), and urea (>99.5%); GenScript Biotech BV (Leiden, The Netherlands): RBD protein (omicron BA.1, C-terminal His-Tag); Honeywell Fluka^TM^ (Seelze, Germany): Ammonium bicarbonate (ABC), Ethane-1,2-dithiol; Promega GmbH (Mannheim, Germany): Peroxidase-conjugated anti-human IgG antibody, PNGase F; Roche Deutschland Holding GmbH (Mannheim, Germany): cOmplete™ Mini EDTA-free protease inhibitor cocktail (from bovine pancreas), DNAse I; Seramun Diagnostika GmbH (Heidesee, Germany): TMB substrate solution; SERVA Electrophoresis GmbH (Heidelberg, Germany): Acrylamide/bis(acrylamide) (30% T, 2.67% C), ammonium persulfate (APS; analytical grade), BlueBlock PF 10×, Coomassie Brilliant Blue G-250, *N*,*N*,*N*′,*N*′-tetramethylethane-1,2-diamine (TEMED, analytical grade), and trypsin (sequencing grade, MS approved); Sigma Aldrich Chemie GmbH (Taufkirchen, Germany): 2-mercaptoethanol (BioUltra), Antifoam Y-30 emulsion, hydroxybenzotriazole (HOBt, ≥97%), imidazole (≥99.5%), *m*-cresol (≥99%), *N*,*N*′-diisopropylcarbodiimide (DIC, ≥98%), polyethylenimin (PEI), and trifluoroacetic acid (TFA, for HPLC, >99%); Surmodics IVD, Inc. (Eden Prairie, MN, USA): StabilZyme^TM^ SELECT; Thermo Fisher Scientific (Waltham, MA, USA): Gibco DMEM, Gibco Fetal Bovine Serum (FBS), Gibco GlutaMAX Supplement, Gibco 100× MEM non-essential amino acids solution, goat anti-human IgA secondary antibody-HRP, penicillin/streptomycin (10,000 U/mL), and SuperBlock^®^ (PBS); VWR International GmbH (Darmstadt, Germany): Acetonitrile (HPLC-gradient grade), diethyl ether, and formic acid (98%).

### 2.1. Serum Collection

Clinical serum samples were obtained from patients hospitalized in the year 2020 (N = 81) with PCR-confirmed SARS-CoV-2 infections (Hospital St. Georg, Leipzig, Germany and Klinikum Chemnitz, Chemnitz, Germany), including 60 samples with information on days after PCR and 36 samples with additional information on symptom onset (Supplement, Table S1). These investigations are part of the analyses in the COVID genetics cohort Leipzig-Chemnitz, which was approved by the Institutional Review Board of Leipzig University (reference numbers 195/20-ek and EK-allg-37/10-1). Control serum samples, collected from 2009 to 2015 and considered negative for SARS-CoV-1/2 and MERS-CoV infections, were obtained from the population-based LIFE-Adult study of the Leipzig Research Center for Civilization Disease (LIFE) [23,24].

### 2.2. Recombinant Proteins Expressed in E. coli

The coding sequence of RBD (C-terminal His_6_-Tag, residues 319–541, NCBI accession YP_00972439) was synthesized, codon-optimized for *E. coli* by GenScript Biotech BV (Leiden, The Netherlands), and cloned into the pET21b(+) vector. The RBD and M-protein were also expressed in *E. coli*, as described previously [25].

### 2.3. Recombinant Proteins Expressed in HEK Cells

A commercial pUC vector harboring the sequence of the proteins (GenScript Biotech BV, Leiden, the Netherlands) was used to clone the sequences of the RBD (318–541 bp) and the full-length M-protein (1–222 bp), together with a C-terminal His-Tag, into the expression vector pHLsec. Subsequent transient transfection and expression of the RBD in HEK293S GnTI− and HEK293T cells were performed as described before [26]. The same procedure was applied for the expression of the M-protein in the stable HEK293S GnTI− cell line, where the medium could be collected throughout expression [25,26]. The proteins were purified by IMAC using a HisTrap^TM^ HP column (GE Healthcare, Solingen, Germany), followed by size-exclusion chromatography (SEC). For additional recovery of M-protein, cells were washed with PBS and then lysed with RIPA buffer (50 mmol/L Tris/HCl, 150 mmol/L NaCl, 1% Triton-X, 0.1% sodium deoxycholate, protease inhibitor (EDTA free), DNAseI, pH 8). The cell suspension was centrifuged, and the supernatant purified by IMAC and SEC.

### 2.4. Glycostaining

Glycoproteins were stained by the Periodic Acid Schiff (PAS) method. The oxidative cleavage of the sugar moieties by periodic acid was followed by staining with dipotassium disulfite and fuchsin (Schiff’s reagent), leading to magenta bands for glycosylated proteins. Afterwards, all proteins were stained with Coomassie Brilliant Blue G250.

### 2.5. Enzyme-Linked Immunosorbent Assay (ELISA)

RBDs from HEK293S GnTI− cells were digested on a centrifugation filter, once with Endoglycosidase F1 (Endo F1, 6 µL of a 1 mg/L solution, overnight, 4 °C) and once with PNGase F (1 h, 37 °C), according to the manufacturer’s instructions. The sample was centrifuged (14,000× *g*, 25 °C, 15 min) to remove the glycans, and the supernatant was used for the following experiments. Medium-binding microplates (Greiner Bio-One, 12xF8, PS, F-bottom) were coated in each well with RBDs expressed in-house in HEK293S GnTI− and HEK293T cells, commercial RBDs (BA.1, BA.4), or deglycosylated RBDs (75 ng) in PBS supplemented with sodium chloride (200 mmol/L), or RBDs (150 ng) expressed in *E. coli* (4 °C, overnight). The RBD protein ELISA was performed as previously described [25]. The same ELISA conditions, except for the addition of 0.05% Tween 20 to the Superblock blocking solution, were used for the deglycosylation study. Significance was tested with an ANOVA test using GraphPad Prism version 10.0.3 (Graph Pad Software, La Jolla, CA, USA).

### 2.6. FASP Digestion

Purified RBD variants were digested by a modified filtration-assisted sample preparation protocol, using pre-conditioned 10-kDa molecular weight filters [27]. All centrifugation steps were performed at 14,000× *g* at 25 °C. Briefly, a solution of 15 µg RBD or 30 µg M-protein was diluted to 335 µL with PBS and centrifuged (15 min). This procedure was repeated once before Endo F1 enzyme was added (6 µL, 1 mg/L) and digested overnight (wet chamber, 37 °C). The digest was reduced and diluted to 50 µL with lysis buffer (60 mmol/L Tris-HCl pH 6.8, 2% (*w*/*v*) SDS, 10% (*w*/*v*) glycerin, 0.1 M DTT) and 200 µL urea solution at 14,000× *g* and centrifuged (15 min). Urea solution (8 mol/L in 0.1 mol/L Tris/HCl solution, 200 µL) was added, centrifuged (for 10 min), and then IAA (100 μL, 50 mmol/L in urea solution) was added to alkylate thiols (for 20 min, in the dark, at RT). Samples were centrifuged (for 10 min). Fresh urea solution (100 μL) was added and centrifuged (for 15 min). This step was repeated twice with urea solution and three times with ABC buffer (100 μL, 0.1 mol/L). Trypsin (1.2 µg, protein/enzyme ratio 25:1) was added, diluted in 40 µL of ABC buffer, incubated overnight (~16 h), and centrifuged (for 10 min) after changing the collection tube of the filtration unit. ABC buffer (50 µL) was added to the filter and centrifuged (for 15 min). The combined filtrates were dried in a vacuum centrifuge and stored at −20 °C.

### 2.7. Lectin Enrichment

Glycosylated peptides were enriched using a recently reported protocol [28]. Here, concanavalin A was used as the lectin due to the high-mannose content of proteins expressed in HEK 293S cells. The glycosylated peptides were deglycosylated using PNGase F or Endo F1, as described above, using ABC buffer (Supplement, Figure S1).

### 2.8. Mass Spectrometry

Digests were separated on a nanoACQUITY Ultra Performance LC™ (Waters Corp., Manchester, UK) coupled online to a Synapt G2-S*i* instrument (Waters, Eschborn, Germany). Peptides corresponding to 100 ng of the initial protein content were trapped online for 6 min on a nanoACQUITY Symmetry C_18_-column (internal diameter (ID) 180 μm, length 2 cm, particle diameter 5 μm) at a flow rate of 5 μL/min of 99% (*v*/*v*) eluent A (water containing 0.1% (*v*/*v*) formic acid) and 1% (*v*/*v*) eluent B (acetonitrile containing 0.1% (*v*/*v*) formic acid). Separation was performed on a BEH 130 C_18_-column (ID 75 μm, length 10 cm, particle diameter 1.7 μm; 35 °C) at a flow rate of 0.3 μL/min, using a gradient consisting of two linear slopes: from 1 to 40% eluent B in 18.5 min and from 40 to 95% eluent B in 5.5 min. The column was equilibrated for 10 min. Samples were ionized using a nanospray PicoTip Emmitter (New Objective, Littleton, CO, USA) at a spray voltage of 3.0 kV. The following source parameters were used: sampling cone of 30 V, source offset of 80 V, source temperature of 100 °C, cone gas flow of 20 L/h, and nanoflow gas pressure of 0.2 bar. A data-dependent acquisition (DDA) approach was created using MassLynx (version 4.2SCN983) and DriftScope (version 2.8). Fragment ions were separated prior to detection by TWIMS. The orthogonal time-of-flight (TOF) pusher voltage and the interval for arriving fragment ion synchronization were used to generate a “wideband enhancement” (WbE) using the Waters standard operating procedure. Full scan MS (*m*/*z* 300–5000) and MS/MS spectra (*m*/*z* 50–5000) were acquired for 0.2 s and 0.4 s, respectively. MS/MS scans were triggered at signal intensities above 1000 counts and acquired once, up to a total ion current (TIC) threshold of 100,000 counts, for a maximum of 0.4 s. Fragmentation was triggered in the trapping region of the ion mobility cell using an *m*/*z*-dependent collision energy ramp from 12.3/17.8 V (*m*/*z* 50, start/end) to 147/183 V (*m*/*z* 5000, start/end). Tandem mass spectra were triggered for the 5 most intense signals using a dynamic exclusion window of ±250 mDa for 6 s and a full cycle ramped wave velocity ranging from 2500 to 400 m/s (start to end), with a constant wave height of 40 V. Ions were trapped at 15 V for 500 μs prior to IMS and extracted at 0 V with an IMS delay of 1000 μs after trap release.

### 2.9. Database Search

Raw DDA files were processed with Mascot Distiller (version 2.8.4.0; Matrix Science Ltd., London, UK) to generate peak lists in mascot generic format (.mgf), which were searched with the Mascot search engine (version 2.8.0) using Mascot Daemon (version 2.6.0). The search used the human reference proteome (loaded on 21 September 2023 https://www.uniprot.org) complemented with sequences of the enzyme Endo F1, the RBD (residues: 319–541 of the S-protein) corresponding to the wildtype and alpha, beta, kappa, gamma, and BA1 VOCs and the M-protein as a *.fasta* file. Search parameters were the following: trypsin considering three missed cleavage sites, cysteine carbamidomethylation (+57.022 Da), methionine oxidation (+15.995 Da), deamidation (+0.984 Da) of asparagine and glutamine, and N-acetylhexosamine (+203.079 Da) and glycan core Hex_5_HexNAc_2_ (+1216.422 Da) on asparagine as variable modifications, peptide tolerance of ±15 ppm and MS/MS ± 0.025 Da. The resulting data files were loaded as a spectral library into Skyline (version 22.2.0.312, https://skyline.ms/project/home/begin.view, MacCoss Lab, Washington, DC, USA, accessed on 28 March 2023) using a score threshold of 0.05 and inclusion of ambiguous matches, and using the following Skyline settings: the background proteome was the database used for the Mascot search, trypsin [KR/P] with a maximum of three missed cleavages, peptides ranging from 8 to 25 residues in length, carbamidomethyl (C), oxidation (M), deamidation (N), Hex_5_HexNAc_2_ (N), and HexNAc (N) as structural modifications, with a maximum of three modifications and one loss for a peptide. For transition settings, the following parameters were selected: “precursor charge: 2, 3, 4”, “y- and b-fragment ion charge 1, 2, 3”, “product ion selection *m*/*z* > precursor to 3 ions”, “special ions N-terminal to proline”, and “auto-select all matching transitions”. For the library ion match, tolerance was set to *m*/*z* 0.5, ‘if a library spectrum is available, pick its most intense ions’ was selected, and 3 product ions were selected from filtered ion charges and types. The instrument *m*/*z* range was set from 50 to 2000, MS1 filtering used the first three precursor ion isotopes, TOF (resolving power 20,000 at *m/z* 400) was used with the acquisition method DDA, using only scans within 5 min of MS/MS IDs, and using high-selectivity extraction. Raw mass spectrometry data and database search results are available on ProteomeXchange under the data set identifier PXD048930. Generated spectral libraries and Skyline documents are available at https://panoramaweb.org/RBD_M_Glyco.url.

### 2.10. Statistical Analysis

Graphs were generated using GraphPad Prism 10.0.3 (Graph Pad Software, La Jolla, CA, USA). Significance with *p*-values—*: 0.01 to 0.05, **: 0.001 to 0.01, ***: 0.0001 to 0.001, ****: <0.0001—was tested with the ordinary one-way ANOVA test using GraphPad Prism. The Receiver Operating Characteristics (ROC) curve analysis was performed using GraphPad Prism after determining the cut-off for best sensitivity and specificity using the Youden Index.

## 3. Results

### 3.1. Confirmation of RBD Glycosylation

**Figure 1:**
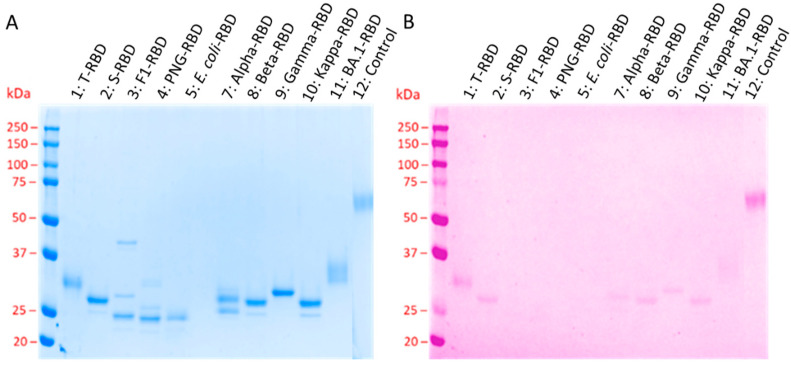
SDS-PAGE of all RBD variants (1 µg protein per lane) stained with Coomassie (**A**) and glycostain (**B**). Lanes 1–5 correspond to the wildtype RBD, lanes 7–11 to the RBD VOCs, and lane 12 to the control protein aGPCR-F1 (16 glycosylation sites). Calculated protein masses: 1: T-RBD > 32 kDa, 2: S-RBD = 31.7 kDa, 3: Endo F1 = 32 kDa, F1-RBD = 29.5 kDa, 4: PNGase F = 35.5 kDa, PNG-RBD = 29.3 kDa, 5: *E. coli* RBD = 26.3 kDa, 7: alpha-RBD = 31.7 kDa, 8: beta-RBD = 31.7 kDa, 9: gamma-RBD 3 = 1.7 kDa, 10: kappa-RBD = 31.7 kDa, and 11: BA.1-RBD > 28.6 kDa.

The purified RBDs were separated by SDS-PAGE and stained with Coomassie Brilliant Blue (Figure 1A lanes 1–11). The major difference between recombinant proteins expressed in bacteria and human cell lines is the absence of post-translational modifications in bacteria, including protein glycosylation and sometimes protein folding. RBDs expressed in HEK293T cells (T-RBD) contain a complex glycosylation pattern, whereas whole SARS-CoV-2 viruses isolated from Vero E6 cell cultures contain predominantly high-mannose glycans at the same asparagine residues [16]. In this study, HEK293S GnTI− cells, which are known to produce high-mannose-type glycoproteins (GlcNAc_2_Man_5_) [29], were used to express RBD proteins from seven different SARS-CoV-2 variants (S-RBD). The putative unmodified wildtype RBD expressed in *E. coli* showed a single sharp band at an apparent molecular weight of ~25 kDa, which was slightly below the calculated molecular weight of 26.3 kDa, but in agreement with previous reports [26]. The band corresponding to the RBD expressed in HEK293S GnTI− cells (S-RBD) appeared at an apparent molecular weight of ~28 kDa, i.e., it shifted by ~3 kDa compared to the *E. coli* RBD, which fits well to two GlcNAc_2_Man_5_ units, each contributing with a molecular weight of ~1217 Da (calculated: 29.3 kDa + 2 × 1.2 kDa) [29,30]. In contrast, the RBD expressed in HEK293T (T-RBD), with an assumed complex glycosylation pattern, showed a diffuse band at ~32 kDa. When the S-RBD was incubated with PNGase F or Endo F1 prior to SDS-PAGE, which completely cleaves the sugar off or leaves a single GlcNAc, the bands shifted to apparent molecular weights of ~24 kDa and ~25 kDa, respectively, which are close to the *E. coli* RBD. Glycosylation of S- and T-RBD and their deglycosylation of the N-glycosylation sites by PNGase F and Endo F1 were confirmed by glycostaining (Figure 1B). The bands of both S- and T-RBDs appeared to be more intense in Coomassie staining than those of the glycoprotein adhesion G protein-coupled receptor F1 (aGPCR-F1) added as a control, but weaker in the glycostain, indicating that the RBDs contained a lower sugar content, i.e., 4 N-glycosylation sites, compared to the control protein, glycosylated at 16 asparagine residues. The very weak bands obtained in the glycostain after deglycosylation (Figure 1B, lanes 3 and 4) could indicate partially remaining N-glycosylation sites or O-glycosylation sites [18,31] that are not cleaved by PNGase F and Endo F1 [32,33]. The bands of the in-house expressed alpha, beta, gamma, and kappa RBDs also appeared at higher apparent molecular weights than the *E. coli* RBD and were also visible in the glycostain, confirming that they were all glycosylated at least at one residue. However, the alpha, beta, and kappa variants displayed a second less intense band at a lower apparent molecular weight that was not visible in the glycostain, indicating that small portions of these proteins were not glycosylated, which was most evident for alpha-RBD, where two bands of similar intensity were obtained at ~26 kDa and ~28 kDa. The commercial BA.1 RBD, which was expressed in HEK293T cells, showed a diffuse band for both staining methods at a similar apparent molecular weight as the T-RBD, indicating the expected complex glycosylation pattern.

### 3.2. Mass Spectrometry Studies

**Figure 2:**
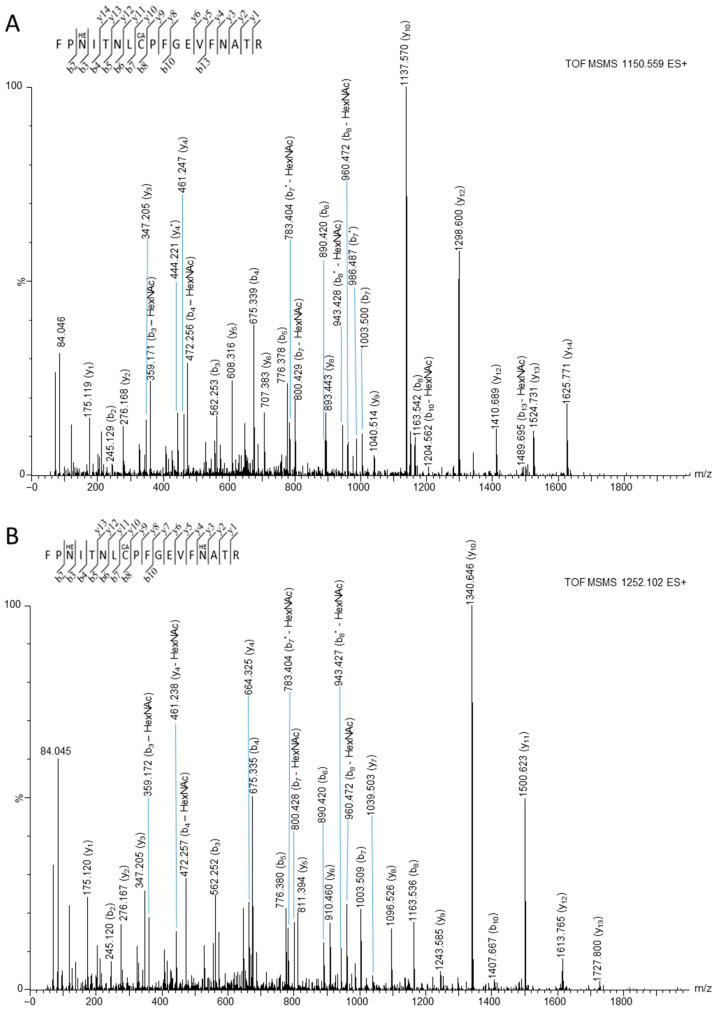

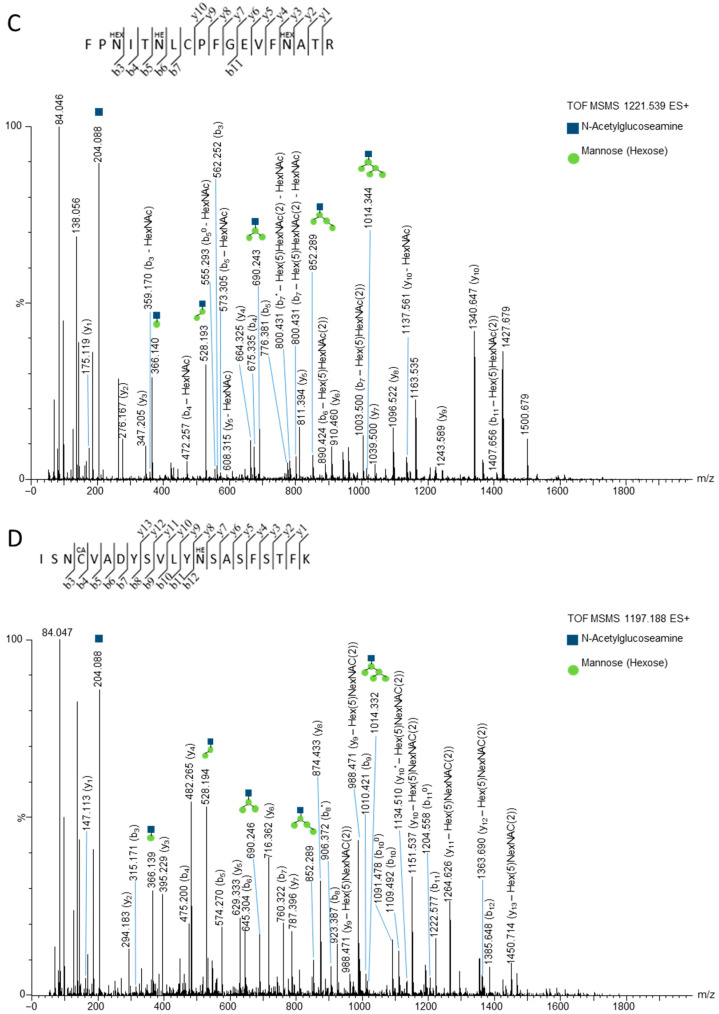
Annotated product ion spectra of glycopeptides FPN[+203.1]ITNLC[+57]PFGEVFNATR (**A**), FPN[+203.1]ITNLC[+57]PFGEVFN[+203.1]ATR (**B**), FPN[+203.1]ITN[+1216.4]LCPFGEVFN[+203.1]ATR (**C**), and ISNC[+57]VADYSVLYN[+203.1]SASFSTFK (**D**) covering residues 329 to 346 (**A**–**C**) and 358 to 378 (**D**) of the wildtype RBD. The peptides were obtained after treating S-RBD first with Endo F1 and then with trypsin. The doubly protonated precursor ions detected at *m*/*z* 1150.559 (**A**) and *m*/*z* 1252.102 (**B**), and the triply protonated precursor ions detected at *m*/*z* 1197.188 (**D**) and *m*/*z* 1221.539 (**C**) were selected for collision-induced dissociation. The fragmentation of the N-linked glycan core is indicated by the corresponding structure (**C**,**D**).

After confirming the N-glycosylation of all RBD variants expressed in HEK cells by glycostaining and glycosidase treatment, the glycosylation sites in RBDs expressed in HEK293S GnTI− cells were mapped by mass spectrometry, as the glycosylation sites have been reported for HEK293T cells but not for HEK293S GnTI− cells. Since N-glycosylated peptides are often missed in standard bottom-up proteomics approaches, the heterogeneity of the glycan structure was reduced by partial cleavage of the sugar moiety with Endo F1 glycosidase, leaving only one GlcNAc unit at the Asn residue, corresponding to a mass shift of 203.079 Da compared to the unmodified sequence. When the RBDs expressed in HEK293 cells were treated first with Endo F1 and then with trypsin, the LC-MS data displayed signals at *m*/*z* 1150.559 and *m*/*z* 1252.102 with a charge state of 2, and *m*/*z* 1197.188 and *m*/*z* 1221.539 with a charge state of 3 for all RBDs expressed in HEK293S GnTl− cells, but not for the wildtype and omicron variant BA.1 (mutated in this region) RBDs expressed in HEK293T cells. The b- and y-series observed in the corresponding tandem mass spectra confirmed the sequence from Phe329 to Arg346 of the wildtype S-protein, including mass increases of ~203.079 Da at both Asn331 and Asn343 (Figure 2A,B). Additionally, a glycosylated peptide was observed with a total mass increase of 1622.582 Da compared to the unmodified sequence, which could be explained by two asparagine residues carrying a GlcNAc and one asparagine residue carrying an N-glycan consisting of two GlcNAc and five mannose units. The y-ion series identified Asn343 (GlcNAc), and the b-ion series identified Asn331 (GlcNAc), suggesting that the remaining Asn334 carries a GlcNAc_2_Man_5_ glycan. However, this glycosylation site could not be confirmed, because only b-ions with a loss of the N-glycan were observed (Figure 2C). Similarly, a peptide with a neutral monoisotopic mass of 3588.522 Da (*m*/*z* 1197.188), corresponding to residues Asn360 to Lys378 of the S-protein, was detected with a carbamidomethylated Cys361 (+57.021Da) and either an additional mass increase of ~1216.4423Da (Figure 2D) or ~203.079 Da (Supplement, Figure S2 XLII–LIII), corresponding to the GlcNAc_2_Man_5_ or the GlcNAc moiety, respectively. The obtained b- and y-signals did not allow the annotation of Asn360 or Asn370 as glycosylated. The GlcNAc and N-linked glycan core were further established by sequential neutral losses of sugar monomers from the b- and y-ions. The mass spectra revealed non-uniform glycosylation patterns for the same peptides (Figure 2A–C), suggesting site heterogeneity [34,35], as Endo F1 did not quantitatively remove the sugar moieties from all residues (Figure 2C,D). The N-glycosylation sites identified for the wildtype RBD were also confirmed for the alpha, beta, gamma, and kappa-RBD VOCs (Supplement Figure S2). For the omicron variant, N-glycosylation was expected at Asn331 and Asn343 in the tryptic peptide FPN[+203.1]ITNLC[+57]PFDEVFN[+203.1]ATR with a neutral monoisotopic mass of 2560.1797 g/mol. However, no signals corresponding to the unglycosylated (2154.0204 g/mol) or modified sequence bearing a GlcNAc moiety at one (2357.0998 g/mol) or two asparagine residues (2560.1797 g/mol) were observed in LC-MS. Even after enriching the tryptic digests with concanavalin A (Supplement, Figure S1), the corresponding peptides could not be detected. In addition, we did not detect the expected peptide in the wildtype T-RBD, perhaps because digestion with Endo F1 is less efficient for sequences with a complex glycosylation pattern.

### 3.3. Cross-Reactivity of Anti-RBD IgG Antibodies against Different VOCs

**Figure 3:**
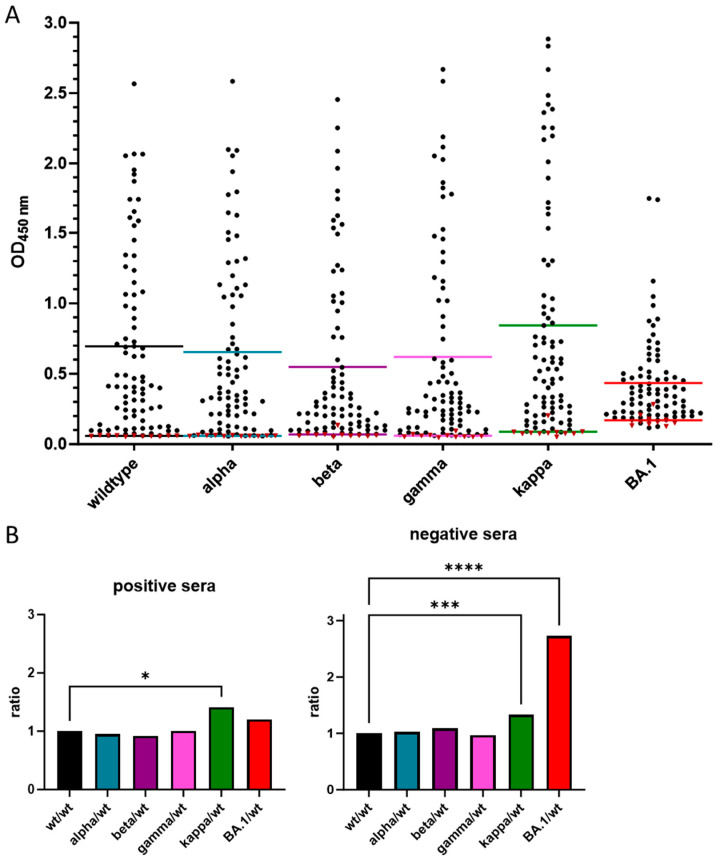
IgG ELISA of the glycosylated VOC RBDs. (**A**) OD_450_ values of 81 positive (black dots) and 10 negative sera (red triangles) used in the IgG ELISA of the different glycosylated VOC RBDs. The mean values are indicated as lines (top: positive, bottom: negative) in the corresponding color (**B**): OD_450_ value ratios of the different VOC RBDs (wildtype: black, alpha: cyan, beta: violet, gamma: magenta, kappa: green, BA.1: red) normalized to the wildtype RBD expressed in HEK293S GnTI− cells of positive and negative sera, with the significance indicated as asterisks (ordinary one-way ANOVA *p* values—*: 0.01 to 0.05 significant, ***: 0.0001 to 0.001 extremely significant, ****: <0.0001 extremely significant).

We tested 81 positive serum samples collected in Germany in the spring of 2020, before the appearance of the alpha VOC, and 10 negative serum samples collected in Germany before 2015 (Supplement, Table S1) using a recently reported anti-RBD IgG ELISA that can test sera for IgG antibodies recognizing the wildtype, alpha, beta, gamma, kappa, and omicron (BA.1) RBD variants [26] (Figure 3). A receiver operating characteristic (ROC) analysis suggested a sensitivity of 96.3% and a specificity of 100% for wt-RBD, whereas typically reduced sensitivity and specificity were observed for alpha (91.4%, 100%), beta (91.4%, 90.0%), gamma (86.4%, 100%), kappa (98.8%, 90.9%), and omicron BA.1 RBDs (92.6%, 81.8%) (Table 1, Supplement Figure S3).

To better illustrate how mutations in the VOC RBDs affect their recognition by IgG antibodies in sera collected from patients infected before the appearance of the first VOCs in Germany, the ratios of the OD_450_ values obtained for each VOC RBD to the wt-RBD were calculated for all serum samples (Supplement Figure S4). In general, mutations in the VOCs altered IgG recognition, with ratios typically changing in the order of appearance of the VOCs, which also corresponded to an increase in the number of mutation sites. The average of all ratios of each VOC RBD altered only slightly, except for a significant increase in the kappa-RBD (*p* = 0.0206). This unexpectedly indicated better recognition of kappa-RBD than wt-RBD, which was mostly associated with ~15 samples with ratios of ~1.5 or greater. Interestingly, there was no clear trend in the order of the VOC RBD recognition for individual samples, as the highest and lowest ratios were typically observed for different serum samples. While the mutations in the alpha-RBD did not affect IgG recognition in the control sera, the beta, gamma, kappa, and BA.1-RBDs showed higher ratios, indicating increasing cross-reactivity. When the average ratios of all control samples were considered for each VOC RBD, the increase was statistically significant only for the kappa (*p* = 0.0001) and BA.1 (*p* < 0.0001) RBD (Figure 3).

### 3.4. Effect of Glycosylation of wt-RBD on IgG Recognition

Previously, we noticed that wt-RBD expressed in *E. coli* showed a much worse sensitivity and specificity than S-RBD in the IgG ELISA [25], which was attributed to the lack of protein glycosylation and maybe improper protein folding. Many studies have shown the importance of glycosylation for infectivity and immune evasion, which might also affect antibody recognition. Thus, wildtype S- and T-RBDs, expressed in HEK293S GnTI− and HEK293T cells, respectively, were compared with their enzymatically deglycosylated versions and *E. coli* RBD. Glycoproteins expressed in HEK293S GnTI− and HEK293T cells are known to contain high-mannose (GlcNAc_2_Man_5_) and complex glycosylation patterns, respectively, whereas whole SARS-CoV-2 viruses isolated from Vero E6 cell cultures contain predominantly high-mannose glycans at the same asparagine residues [16].

**Figure 4:**
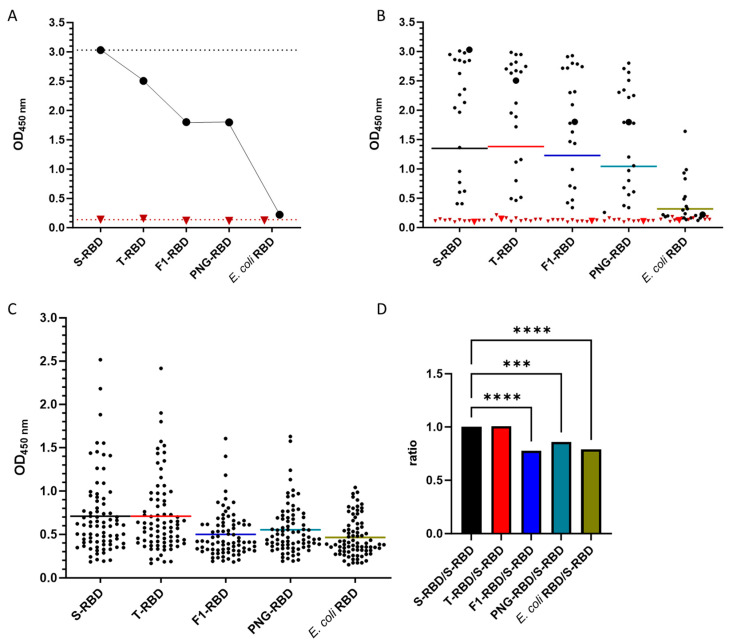
IgG antibody recognition of glycosylated, deglycosylated, and unglycosylated wildtype RBDs. (**A**): OD_450_ values of a positive (black, circle) and a negative pool (red, triangle) used in the IgG ELISA of the different glycosylated RBDs. The values of the S-RBD are indicated as dotted lines in the corresponding serum pool color. (**B**): OD_450_ values of twenty positive (black) and ten negative sera (dark red) and the positive (large black dots) and negative pools (large red triangles) used in the IgG ELISA of the different glycosylated RBDs. The mean values are indicated as lines in the following colors: S-RBD (black), T-RBD (red), F1-RBD (blue), PNG-RBD (cyan), and *E. coli* RBD (green) RBDs. (**C**): OD_450_ values of 81 positive sera used in the IgG ELISA of the different wildtype RBDs. The mean values are indicated as lines in the above-mentioned color. (**D**): Shown are the mean values for all 81 serum samples of the OD_450_ values of S-RBD (black), T-RBD (red), F1-RBD (blue), PNG-RBD (cyan), and *E. coli* RBD (green) normalized to the OD_450_ value of the wildtype RBD expressed in HEK293S GnTI− cells (S-RBD). Significance is indicated by ordinary one-way ANOVA *p* values: ***: 0.0001 to 0.001 extremely significant, ****: <0.0001 extremely significant.

A positive pool strongly recognized both T- and S-RBDs similarly well with OD_450_ values of ~3.0 and ~2.5, respectively, whereas the negative pool provided a much lower OD_450_ values of ~0.1 (Figure 4A). Deglycosylation with PNGase F and Endo F1 significantly reduced the recognition of the positive pool to ~1.8, while the OD_450_ values of the negative pool remained stable. *E. coli* RBD was not recognized at all. Similar trends were observed for the average of 20 positive and 10 negative serum samples tested first (Figure 4B) and for the full set of 81 positive serum samples (Figure 4C). To facilitate comparison of the recognition of RBD variants by individual sera, the OD_450_ value of each RBD was normalized by dividing it by the corresponding OD_450_ value of the S-RBD (Supplement Figure S5). The mean normalized values of the positive sera of T-RBD were around 1, indicating that the different glycan structures had no significant effect on the IgG recognition (Figure 4). The normalized values for deglycosylated PNG- and F1-RBDs (*p* = 0.0009, *p* < 0.0001) were significantly lower, with F1-RBD similar to *E. coli* RBD (*p* < 0.0001). Although these significant changes appear to be minor, the reduced OD_450_ values in positive sera worsened the assay sensitivity significantly. This suggests that glycosylation of the RBD is important for the recognition of IgG antibodies produced in response to a SARS-CoV-2 infection, whereas the type of glycosylation has only a minor influence and is most likely not critical for an ELISA.

**Figure 5:**
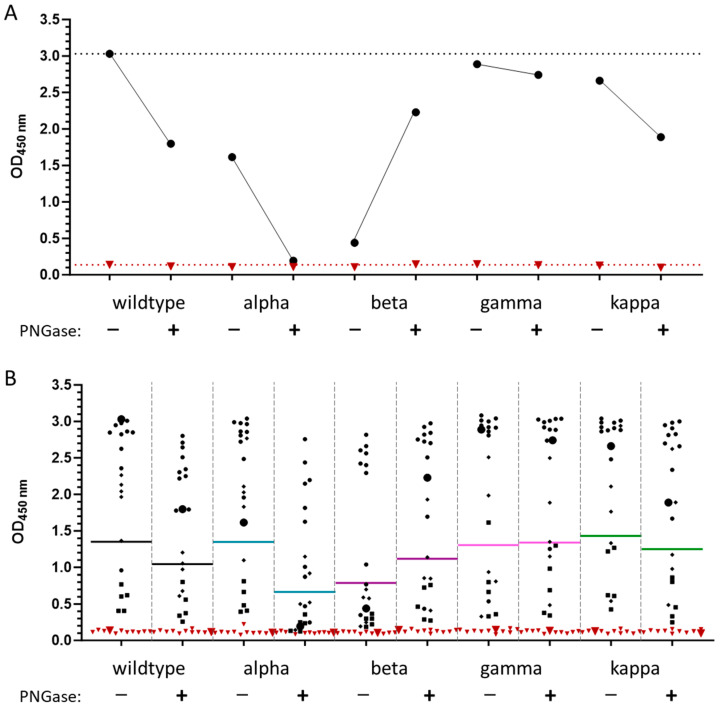
IgG antibody recognition of glycosylated and deglycosylated VOC RBDs. (**A**): OD_450_ values of a positive (black, circle) and a negative pool (dark red, triangle) used in the IgG ELISA without (−) and with PNGase treatment (+) of the VOC RBDs. The values obtained with the positive pool for the glycosylated and the corresponding deglycosylated RBDs are connected by a line. The dotted line indicates the OD_450_ value of wildtype RBD. (**B**): OD_450_ values of twenty positive sera (black) and ten negative sera (dark red, triangle) used in the IgG ELISA of the VOC RBDs. The mean values are indicated as horizontal lines using the following colors: black for wildtype, cyan for alpha, purple for beta, magenta for gamma, and green for kappa. Positive sera were selected based on ELISA data obtained for the wildtype S-RBD: dots indicate sera with high OD_450_ values (N = 10), diamonds indicate sera strongly affected by protein deglycosylation (N = 5), squares indicate sera not affected by deglycosylation (N = 5), and triangles indicate negative sera (N = 10). The positive pool (black) and negative pool (dark red) are marked with enlarged circles and triangles, respectively.

For further clarification, the negative and positive pools (Figure 5A), 10 negative (triangles), and 20 positive (due to the limited protein quantities) samples with the OD_450_ values obtained in the ELISA, showing high values (N = 10, dots) and those strongly affected (N = 5, diamonds) or slightly affected (N = 5, squares) by deglycosylation were selected and tested for all VOCs before and after deglycosylation with PNGase F (Figure 5B). Deglycosylation of VOCs typically decreased the OD_450_ values of the positive pool (Figure 5A) and the mean values (Figure 5B), although the opposite trend was observed for the beta variant. It should be noted that the small number of samples does not allow for more general conclusions, especially as all samples were obtained from patients infected with the original SARS-CoV-2 strain that spread in Europe. Furthermore, the glycosylated beta variant showed the worst results, and although it improved after deglycosylation, it did not reach the level of the wildtype RBD.

### 3.5. Membrane Protein

**Figure 6:**
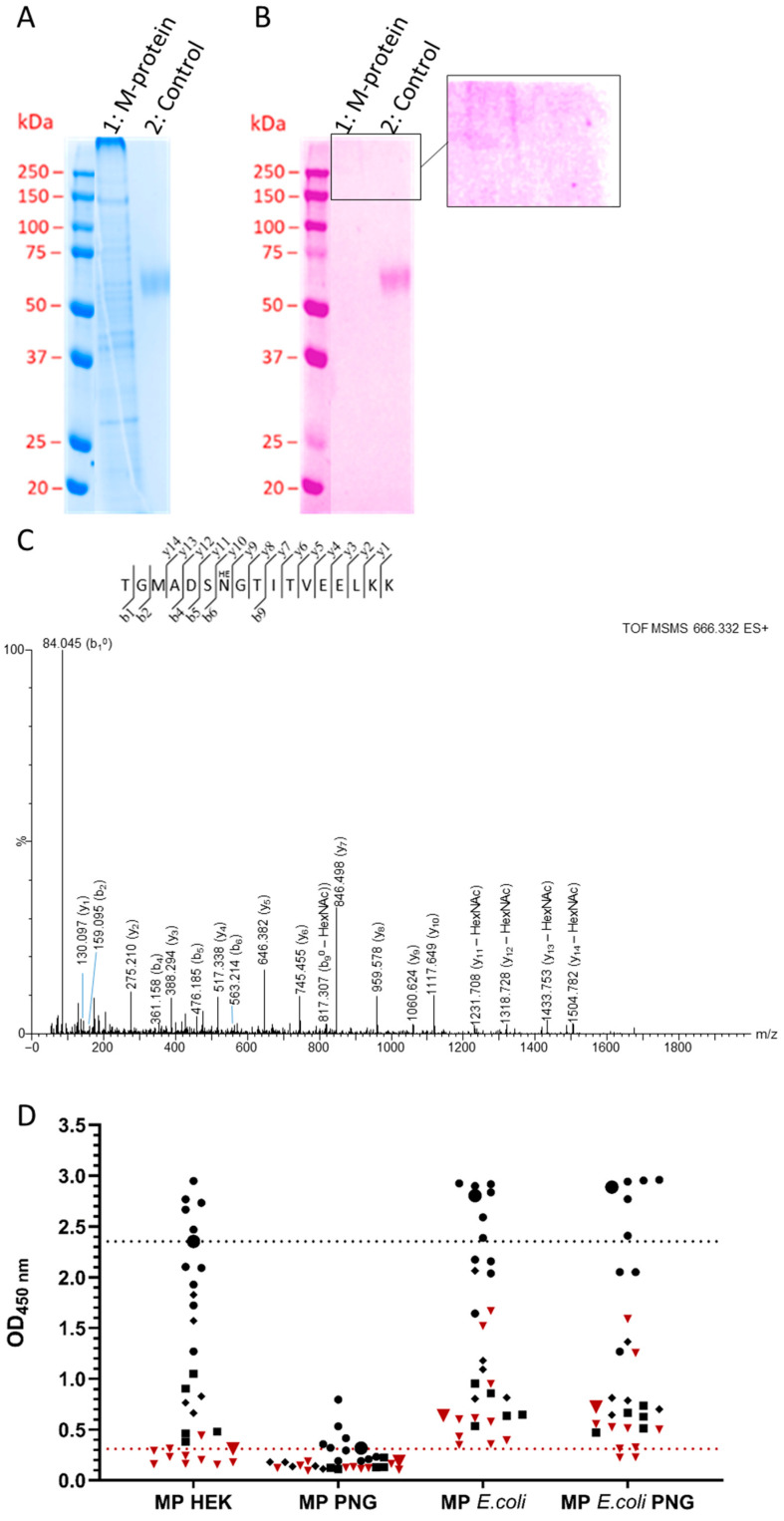
Characterization of purified M-protein expressed in a stable HEK293S GnTI− cell line. SDS-PAGE of SARS-CoV-2 M-protein (1 µg) stained with Coomassie Brilliant Blue (**A**) and glycostain (**B**). Lanes 1 and 2 correspond to the M-protein and the control glycoprotein aGPCR-F1, respectively. The M-protein was detected only in the upper marked and zoomed area of the gel, indicating multimers. Calculated protein masses: M-protein 30.5 kDa (monomer, 29.3 + 1.2 kDa). (**C**): Annotated fragment ion spectrum of the triply protonated glycopeptide TGMADSN[+203.0794]GTITVEELKK (*m*/*z* 666.332) covering residues Met1-Lys15 of the M-protein digested with trypsin, enriched with concanavalin A, and then treated with Endo F1 and PNGaseF. (**D**): OD_450_ values of twenty positive sera (black, circle) and ten negative sera (dark red, triangle) tested in the IgG ELISA coated with M-protein expressed in HEK293S cells (MP HEK) and PNGase-treated M-protein present in a HEK293S lysate (MP PNG), as well as M-protein expressed in *E. coli* before (MP *E. coli*) or after PNGase-treatment (MP *E. coli* PNG, 18 positive sera). Positive sera were selected based on ELISA data obtained for the wildtype S-RBD: dots indicate sera with high OD450 values (N = 10), diamonds indicate sera highly affected by protein deglycosylation (N = 5), squares indicate sera not affected by deglycosylation (N = 5), and triangles indicate negative sera (N = 10). The positive and negative pools are indicated as enlarged black circles and dark red triangles, respectively. The dotted lines indicate the OD_450_ values of the pools for the HEK M-protein.

Having observed that N-glycosylation of the RBD is important for recognition by IgG antibodies in the sera of SARS-CoV-2-infected individuals, we also examined the M-protein with its proposed single N-glycosylation site. In a previous study, we showed that the M-protein expressed in *E. coli* provided only low sensitivity (41%) and moderate specificity (88%), which is not suitable for the serological detection of SARS-CoV-2 infection. Expression and affinity purification of the lysate provided the M-protein in low yields (100 µg). When the monomeric M-protein was collected on SEC, it reassembled into the multimer in solution. This is most likely due to the physicochemical properties of the M-protein, as it dimerizes either with itself or with the envelope or nucleocapsid protein in the virus membrane [6]. In addition, the M-protein was contaminated with several HEK cell proteins, which were identified by mass spectrometry using an in-solution tryptic digest of the purified protein. The SDS-PAGE showed multiple bands along the entire lane, indicative of M-protein oligomers and contaminating HEK cell proteins (such as HNRPL_HUMAN, DDX42_HUMAN, and LMNA_HUMAN), whereas the glycostain showed only a faint pink colored smear at the top of the lane, which may indicate multimeric glycosylated forms of the M-protein that were unable to migrate into the gel, and no band was observed at the expected position of the monomeric M-protein at ~30 kDa (Figure 6A,B). It is surprising to see these multimeric forms on SDS-PAGE when the monomeric fraction of the M-protein from SEC was loaded onto the gel. One explanation could be that the M-protein formed larger complexes (micelles) with SDS, which migrated very slowly.

The very faint band intensities in the glycostain suggest a low glycosylation degree. When the protein was digested first with Endo F1 and then with trypsin, a weak signal was observed at *m*/*z*666.332, corresponding to the monoisotopic mass of a tryptic peptide (1995.967 g/mol), likely containing a GlcNAc unit at Asn7 of the peptide sequence (Asn5 of the M-protein sequence), i.e., TGMADSN[+203.1]GTITVEELKK. However, a tandem mass spectrum was not triggered in DDA mode, as the signal intensity was too low. Thus, a modified FASP approach was applied to enrich for glycosylated peptides [28], which indeed allowed for the enrichment and subsequent recording of a tandem mass spectrum, confirming the peptide TGMADSNGTITVEELKK, with a mass shift suggesting a single GlcNAc unit (Figure 6C). The glycosylation site is most likely at Asn7, but the absence of glycosylated y- and b-signals and the detection of only deglycosylated y-signal series did not allow the confirmation of the glycosylation site Asn5. When the sample additionally treated with PNGase F was analyzed, a tandem mass spectrum was triggered for a signal at *m*/*z* 897.949 (z = 2), which unambiguously identified the peptide TGMADSN[+1]GTITVEELKK (Supplement, Figure S6). The mass shift indicating deamidation of Asn7to give Asp7, confirmed by b_6_-, b_7_-, y_9_-, and y_10_-signals, suggests deglycosylation of Asn(GlcNAc)7 by PNGase F.

When the glycosylated M-protein was used in an ELISA to screen 20 serum samples from infected patients for IgG antibodies, we observed an improved sensitivity of 95.0% and a specificity of 100%. Upon deglycosylation with PNGase, the OD_450_ values of the positive samples strongly decreased, whereas the OD_450_ values of the negative control samples were not affected, reducing the sensitivity to 65% and specificity to 90%. Both results differ significantly from the M-protein expressed in *E. coli*, where both the patient and negative control samples provided high OD_450_ values, limiting the assay specificity to only 70%, with a sensitivity of 95% (Figure 6D and Supplement, Figure S7), which was not altered upon PNGase treatment, suggesting that the M-proteins expressed in *E. coli* adopt a different structure or oligomerization state compared to the deglycoslated M-proteins expressed in HEK293 cells.

## 4. Discussion

The wildtype RBD and all five tested VOC-RBDs expressed in HEK293T and HEK293S GnTI− cells were N-glycosylated based on their higher apparent molecular weights in SDS-PAGE and increased mobility upon treatment with PNGase F or Endo F1, as well as positive and negative glycostains before and after deglycosylation (Figure 1). Mass spectrometry confirmed the expected N-glycosylation sites at Asn331 and Asn343 for all investigated RBDs expressed in HEK293S GnTI− cells. Additionally, Asn334 and probably Asn360 or 370 expressed in HEK293S GnTI− cells were glycosylated. N-glycosylation of Asn334 was previously reported for RBDs expressed in insect cells [4]. Sera obtained from patients infected with the original SARS-CoV-2 strain in early 2020 recognized most of the other VOC RBDs tested very well, although sensitivity and specificity decreased in the order of appearance of the VOCs with BA.1, showing the worst results, consistent with mutations to escape an immune response acquired during previous infections. Interestingly, the kappa-RBD was typically better recognized than the wildtype-RBD, with a ~28% increase in the mean (Table 1), but the specificity was still worse due to the elevated background in negative control sera. In conclusion, the wildtype RBD provided the best test results for sera obtained from patients infected with SARS-CoV-2 variants very close to the wildtype RBD.

The question remains whether antibody binding depends on the mutation sites, which would be most obvious for the BA.1 variant with 15 mutation sites, although recognition of the kappa variant with only two mutations (E484Q, L452R) was also significantly reduced. Since the epitopes and the concentrations of the corresponding antibodies will vary among serum samples from different patients, it is impossible to answer this question without a detailed epitope characterization for each serum, which was not the intention of the current study, and would cover mostly linear epitopes. Previous studies have already shown a large diversity of linear epitopes among patient sera without a dominant epitope characteristic for all patients [36,37]. Nevertheless, mutations in common epitope regions will have a strong effect on individual sera, but this cannot be generalized.

The second aspect to consider for antibody binding is the N-glycosylation pattern of residues Asn331, Asn334, and Asn343 in the RBD, which appears to be characteristic for all VOCs studied here, up to the kappa variant. The two glycosylation sites Asn331 and Asn343 were confirmed by tandem mass spectrometry for the recombinant wildtype RBD expressed in HEK293S GNTI− cells (Figure 2A,B), in full agreement with previous reports [4,16,38,39,40], and the alpha, beta, gamma, and kappa RBDs expressed in HEK293S GnTI− cells (Supplement, Figure S2) [12,18,19]. Additionally, glycosylation at Asn334, previously only observed in insect cells [4], and probably at Asn360 or Asn370, was confirmed (Figure 2C,D, Supplement, Figure S2) for all RBDs, up to the kappa variant. Expectedly, these residues were not glycosylated in the *E. coli* RBD. We were unable to detect the corresponding glycopeptide in the omicron variant BA.1, although the N-glycosylation sites Asn331 and Asn343 were previously confirmed for BA.1 [18]. This might be related to the Gly339Asp mutation, which could reduce the ionization efficiency in positive ion mode and thus analytical sensitivity, or to its complex glycan structure resulting from expression in HEK293T, which may not be well cleaved by Endo F1. This is further supported by the fact that we also could not detect this peptide in the digested wildtype RBD expressed in HEK293T cells.

The different glycosylation patterns obtained in HEK293T and in HEK293S GnTI− cells, i.e., complex glycosylation and high-mannose patterns, respectively, did not affect antibody binding for the wildtype RBD and most likely will not affect it for other VOC-RBDs with the same glycosylation sites. S1 produced by Vero E6 cells infected with the SARS-CoV-2 WA1 strain or the D614G variant predominantly contain high-mannose-type glycans [16], whereas expression in HEK293 leads to more complex-type N-glycans. To mimic the high-mannose-type glycan structure of infected Vero E6 cells, we expressed the wildtype and all VOC-RBDs in HEK293S GnTI− cells in-house, except for the omicron variant BA.1, which was only available as recombinant protein expressed in HEK293T cells. However, the ELISA data obtained for the wildtype RBD expressed in HEK293T and in HEK293S GnTI− cells were very similar, suggesting that the glycosylation type is not very important in serological assays.

Deglycosylation with PNGase F significantly reduced the recognition in the IgG ELISA to the level of the wildtype RBD expressed in *E. coli* (Figure 4C), which is unglycosylated. Assuming that IgG antibodies recognize the protein but not the sugar, it was expected that deglycosylation would not decrease epitope recognition, or that it might even increase antibody recognition by unmasking epitopes. The observed decrease may indicate structural changes at some epitopes, which could reduce antibody binding. Since the effect on the ELISA varied among serum samples, we hypothesize that the structural changes altered the binding to or exposure of some epitopes, which would explain the observed decrease in OD values in most samples and the increase in some samples. This suggests that N-glycosylated RBDs are required for the serological detection of anti-RBD IgG antibodies, whereas the glycan type seems to be less relevant. This finding confirms a previous report showing that the binding affinity of ACE-2 to the trimeric spike protein was not much affected by the expression system used, i.e., baculovirus-insect, Chinese hamster ovary (CHO), HEK 293E, and HEK 293F cells, despite different glycan structures [41]. However, this study showed that complex glycosylation reduced binding to ACE-2 compared to the high-mannose type. In both cases, complete deglycosylation of the N-glycosylation sites strongly increased ACE-2 binding. Ultimately, the question remains as to whether deglycosylation allows the virus to partially escape the immune system and simultaneously increase infectivity, since our in vitro study showed that IgG antibodies are less able to recognize deglycosylated RBD, which is associated with increased binding to ACE-2 [41]. However, previous work showed that suppression of the key glycosyltransferase of the S-protein increases the binding affinity of neutralizing antibodies, and complete deletion of sites N331 and N343 drastically reduces pseudo-typed virus entry [13,17]. Given our results suggesting that IgG antibodies induced by N-glycosylated RBDs are less efficient at recognizing RBDs lacking one or both N-glycosylation sites, mutations in either N-glycosylation site would likely result in more severe infections, even in previously infected or vaccinated individuals. It is tempting to speculate that the improved binding to ACE-2 and reduced binding to vaccination- or infection-induced antibodies against the glycosylated RBD may suggest a promising therapeutic approach, using deglycosylated RBDs to inhibit infection of cells without interfering with the immune response. However, further studies are necessary to verify this hypothesis. In this context, it is interesting to note that the deglycosylated beta and gamma RBDs bind better or as well as the corresponding glycosylated versions. Both RBDs contain the same mutations at sites N501, E484, and K417, of which only K417 is not present in the alpha and kappa RBDs, which may indicate that this mutation has a strong effect on antibody recognition and may represent an escape mutation. However, no valid conclusion can be drawn due to the small number of samples tested.

Based on the above observation that glycosylated RBDs expressed in HEK293S cells provide much better serological data than deglycosylated and unglycosylated RBDs, we also reconsidered the M-protein for an IgG-ELISA to see if its expression in HEK293S cells would improve the accuracy of the assay compared to the M-protein expressed in *E. coli* [25]. To our knowledge, glycosylation sites have not been reported for the SARS-CoV-2 M-protein [4], although in silico methods have suggested asparagine residues 5, 21, 41, 43, 117, 121, 203, and 216 as potential N-glycosylation sites [42,43]. The weak bands in glycostain (Figure 6B) suggest either very low glycosylation levels at different residues or, more likely, glycosylation of a single residue, which is also supported by data from the highly homologous SARS-CoV-1 M-protein and the NetNGlyc web tool, which both suggest Asn5 as the sole glycosylation site [44,45]. We were able to confirm this N-glycosylation site by tandem mass spectrometry after enrichment of a tryptic digest (Figure 6C), but this does not exclude that we missed other glycosylation sites. Interestingly, the glycosylated M-protein provided a good sensitivity of ~95% in the IgG ELISA, which decreased to ~65% after deglycosylation. This is comparable to previous data using the unglycosylated M-protein expressed in *E. coli* [25], although the data cannot be directly compared due to the use of different sets of serum samples. However, the reduced sensitivity of the assay does not properly reflect the significant decrease in OD values from approximately 3 to 1 upon PNGase treatment. In contrast, the unglycosylated M-protein expressed in *E. coli* was strongly recognized by both positive and negative sera, which was expected to be unaffected by PNGase treatment, resulting in similarly low assay sensitivity in both cases. Most likely, M-proteins expressed in HEK293 cells and *E. coli* adopt different structures or oligomerization states due to the sugar moiety, which may also improve solubility. Deglycosylation will affect these properties of the HEK protein by affecting its binding to the surface of the microtiter plate and thus shielding the epitopes, perhaps by oligomerization. Alternatively, deglycosylation may induce strong structural changes that prevent recognition of structural or buried linear epitopes. Nevertheless, it may be interesting to reconsider the M-protein for the serological detection of SARS-CoV-2 infection due to its highly conserved structure, as it may allow detection of SARS-CoV-2 infection independent of mutations in the RBD or S-protein, which are characteristic of escape VOCs. Such variants may require testing of multiple RBDs in the future.

## 5. Conclusions

The current study confirmed the known glycosylation sites at asparagine 331, 334, and 343 and identified an additional glycosylation site at either asparagine 360 or 370 of the SARS-CoV-2 RBD for several VOCs and for the SARS-CoV-2 M-protein at asparagine 5 when expressed in HEK293S GnTI− cells. Interestingly, the glycosylated proteins (RBDs and M-protein) were much better detected in the IgG ELISA than the corresponding deglycosylated proteins, resulting in a much better assay sensitivity for serological detection. At least for the wildtype RBD, the type of N-glycosylation appeared to be less important, since wildtype RBDs expressed in HEK293T cells (complex glycan type) and HEK293S cells (high-mannose glycan type) provided very similar ELISA results. Since the glycosylation sites in alpha, beta, kappa, and omicron BA.1 were identical to the wildtype RBD, the different sensitivities and specificities observed for the IgG ELISA appear to be mostly, if not entirely, related to the mutation sites. The reduced antibody binding to deglycosylated RBD in the patient sera reported here, together with the previously reported improved binding of deglycosylated RBD to the human ACE-2 receptor [41], may suggest a promising strategy for the development of unglycosylated analogs of RBD with improved binding to ACE-2 without cross-reactivity to anti-SARS-CoV-2 antibodies. Unexpectedly, the glycosylated M-protein expressed in HEK293S GnTI− cells provided a good assay sensitivity. The low number of samples tested do not allow for general conclusions, but it might be worth developing a serological assay to detect SARS-CoV-2 infections. However, the expression yields were rather low, and the M-protein was difficult to purify, which may generally limit its application in serological assays.

## Figures and Tables

**Table 1:** Sensitivity, selectivity, cutoff, and mean values of the IgG ELISAs from the different VOC RBDs. Associated plots and ROC curves are in the Supplement Figure S3. (wildtype: black, alpha: cyan, beta: violet, gamma: magenta, kappa: green, BA.1: red).

Protein		Cutoff	Sensitivity	Specificity	Mean Value	
			[%]	[%]	Positive	Negative
	Wildtype	>0.067	96.30	100.00	0.659 ± 0.63	0.058 ± 0.003
	Alpha	>0.067	91.36	100.00	0.653 ± 0.61	0.060 ± 0.003
	Beta	>0.078	91.36	90.91	0.548 ± 0.60	0.069 ± 0.021
	Gamma	>0.094	86.42	100.00	0.619 ± 0.67	0.060 ± 0.012
	Kappa	>0.090	98.77	90.91	0.843 ± 0.78	0.088 ± 0.037
	BA.1	>0.182	92.59	81.82	0.434 ± 0.31	0.170 ± 0.042

## Data Availability

All data generated or analyzed during this study are included in this published article, its Supplementary Materials, and on Panorama (https://panoramaweb.org/RBD_M_Glyco.url).

## Supplementary Materials

The following supporting information can be downloaded at: https://www.mdpi.com/article/10.3390/biology13040207/s1, Table S1: Clinical parameters of patient serum samples; Figure S1: Scheme of the FASP method and lectin enrichment for MS analysis; Figure S2: Tandem mass spectra of the relevant glycopeptides from VOC RBDs; Figure S3: IgG ELISA of VOC RBDs; Figure S4: Normalized IgG ELISA values of different VOC RBDs; Figure S5: Normalized IgG ELISA values of different glycosylated RBDs; Figure S6: Tandem mass spectrum of peptide TGMADSN[+1]GTITVEELKK; Figure S7: M-protein ELISA and ROC curves.
