# Supplementary material for: Influence of Mutations and N-Glycosylation Sites in the Receptor-Binding Domain (RBD) and the Membrane Protein of SARS-CoV-2 Variants of Concern on Antibody Binding in ELISA
Source: Biology (Basel). 2024 Mar 23;13(4):207. doi: 10.3390/biology13040207 (PMC11047955; doi:10.3390/biology13040207)
Supplement: Supplementary file 1 [file biology-13-00207-s001.zip › biology-2892930-supplementary.pdf]

Supplementary Material

Influence of mutations and N-glycosylation sites in the receptor-binding domain (RBD) and the membrane protein of SARS-CoV-2 variants of concern on antibody recognition by ELISA.

Mandy Schwarze[1,2], Daniela Volke[1,2], Juan Camilo Rojas Echeverri[1,2], Robin Schick[1,2], Nicole Lakowa [3], Thomas Grünewald[3], Johannes Wolf[4,5], Stephan Borte[4,5], Markus Scholz[6,7], Andor Krizsan[1,2], and Ralf Hoffmann[1,2,*]

1 Institute of Bioanalytical Chemistry, Faculty of Chemistry and Mineralogy, Universität Leipzig, 04103 Leipzig, Germany
2 Center for Biotechnology and Biomedicine, Universität Leipzig, 04103 Leipzig, Germany
3 Clinic for Infectious Diseases and Tropical Medicine, Klinikum Chemnitz, 09113 Chemnitz, Germany.
4 Department of Laboratory Medicine, Hospital St. Georg gGmbH, 04129 Leipzig, Germany
5 ImmunoDeficiencyCenter Leipzig (IDCL), Jeffrey Modell Diagnostic and Research Center for Primary Immunodeficiency Diseases, Hospital St. Georg gGmbH, 04129 Leipzig, Germany.
6 Institute for Medical Informatics, Statistics and Epidemiology, Universität Leipzig, 04107 Leipzig, Germany
7 LIFE Research Center of Civilization Diseases, Universität Leipzig, 04103 Leipzig, Germany

* Corresponding author: bioanaly@rz.uni-leipzig.de

Table of Contents

Table S1: Clinical parameters of patient serum samples 2
Figure S1: Scheme of the FASP method and lectin enrichment for MS analysis 5
Figure S2: Annotated product ion spectra for all tested RBDs 6
Figure S3: IgG ELISA of VOC RBDs 20
Figure S4: Normalized IgG ELISA values of different VOC RBDs.......................................21
Figure S5: Normalized IgG ELISA values of different glycosylated RBDs............................23
Figure S6: Annotated product ion spectrum of TGMADSN[+1]GTITVEELKK 24
Figure S7: M-protein ELISA and ROC curves..25

Table S1: Clinical parameters of all patient serum samples included in the current study. The samples are sorted by days after symptom onset, days after PCR, and SARS-CoV-2 positive and negative samples, as previously reported [1]. Samples were used to test the glycosylated VOCs and wt-RBDs in the IgG ELISA, whereas the small sample set was used for the deglycosylation study of RBD- and M-proteins. The order is the same as shown in Figures S2 and S3.

Name	Origin	Sex	Age	Days after symptom onset	Days after PCR	IgG VOCs	IgG different glycosylated wt-RBDs	*IgG small sample set*
SG4	St. Georg 2020	m	25	**3**	--	x	x	
SG16	St. Georg 2020	m	24	**23**	51	x	x	
SG13	St. Georg 2020	f	51	**31**	31	x	x	
SG37	St. Georg 2020	f	64	**33**	0	x	x	x
SG15	St. Georg 2020	f	65	**33**	32	x	x	
SG36	St. Georg 2020	f	40	**34**	31	x	x	x
SG7	St. Georg 2020	f	90	**34**	31	x	x	x
SG31	St. Georg 2020	f	39	**34**	32	x	x	
SG29	St. Georg 2020	m	49	**34**	32	x	x	
SG34	St. Georg 2020	m	42	**34**	29	x	x	
SG22	St. Georg 2020	f	52	**35**	29	x	x	
SG9	St. Georg 2020	f	58	**35**	0	x	x	
SG32	St. Georg 2020	f	34	**35**	0	x	x	x
SG10	St. Georg 2020	m	55	**36**	1	x	x	
SG3	St. Georg 2020	f	64	**37**	34	x	x	x
SG11	St. Georg 2020	m	59	**38**	33	x	x	
SG28	St. Georg 2020	m	15	**38**	0	x	x	
SG27	St. Georg 2020	f	39	**38**	33	x	x	
SG19	St. Georg 2020	f	39	**38**	33	x	x	
SG1	St. Georg 2020	m	35	**38**	0	x	x	x
SG6	St. Georg 2020	m	78	**40**	44	x	x	
SG33	St. Georg 2020	f	54	**42**	36	x	x	x
SG2	St. Georg 2020	f	54	**43**	42	x	x	x
SG17	St. Georg 2020	m	28	**44**	--	x	x	
SG12	St. Georg 2020	m	54	**44**	42	x	x	
SG8	St. Georg 2020	m	47	**44**	39	x	x	
SG23	St. Georg 2020	m	63	**45**	30	x	x	x
SG24	St. Georg 2020	m	17	**45**	--	x	x	
SG26	St. Georg 2020	f	54	**47**	44	x	x	x
SG30	St. Georg 2020	m	50	**48**	44	x	x	
SG14	St. Georg 2020	m	53	**49**	55	x	x	
SG20	St. Georg 2020	m	63	**51**	30	x	x	
SG18	St. Georg 2020	f	64	**53**	44	x	x	
SG5	St. Georg 2020	f	38	**54**	52	x	x	

SG25	St. Georg 2020	m	52	**55**	33	x	x	
SG21	St. Georg 2020	m	42	**55**	46	x	x	x
SG68	St. Georg 2020	f	80	--	**0**	x	x	
C13.1	Chemnitz 2020	f	67	--	**0**	x	x	
C26	Chemnitz 2020	m	78	--	**0**	x	x	
C28	Chemnitz 2020	f	93	--	**0**	x	x	
C41	Chemnitz 2020	--	--	--	**0**	x	x	
C14.1	Chemnitz 2020	m	68	--	**1**	x	x	
C32.1	Chemnitz 2020	f	79	--	**1**	x	x	
C33	Chemnitz 2020	--	--	--	**1**	x	x	
C30	Chemnitz 2020	m	76	--	**2**	x	x	
C37.1	Chemnitz 2020	m	37	--	**2**	x	x	x
C35	Chemnitz 2020	m	80	--	**3**	x	x	
C40	Chemnitz 2020	m	75	--	**4**	x	x	
C27	Chemnitz 2020	f	88	--	**5**	x	x	
C31	Chemnitz 2020	m	82	--	**5**	x	x	
C23	Chemnitz 2020	f	62	--	**6**	x	x	
C13.2	Chemnitz 2020	f	67	--	**10**	x	x	
C38.2	Chemnitz 2020	f	75	--	**15**	x	x	
C36	Chemnitz 2020	m	81	--	**16**	x	x	x
C42	Chemnitz 2020	--	--	--	**19**	x	x	
C39	Chemnitz 2020	f	71	--	**20**	x	x	
C21	Chemnitz 2020	f	62	--	**22**	x	x	
C18	Chemnitz 2020	m	67	--	**25**	x	x	
C19	Chemnitz 2020	f	64	--	**25**	x	x	
C11	Chemnitz 2020			--	**28**	x	x	
C43	Chemnitz 2020	--	--	--	**--**	x	x	
C44	Chemnitz 2020	--	--	--	**--**	x	x	
C45	Chemnitz 2020	--	--	--	**--**	x	x	
C46	Chemnitz 2020	--	--	--	**--**	x	x	
C47	Chemnitz 2020	--	--	--	**--**	x	x	x
C48	Chemnitz 2020	--	--	--	**--**	x	x	x
SG69	St. Georg 2020	f	45	--	**--**	x	x	
C49	Chemnitz 2020	--	--	--	**--**	x	x	x
C50	Chemnitz 2020	--	--	--	**--**	x	x	x
C51	Chemnitz 2020	--	--	--	**--**	x	x	
C52	Chemnitz 2020	--	--	--	**--**	x	x	x
C53	Chemnitz 2020	--	--	--	**--**	x	x	
C54	Chemnitz 2020	--	--	--	**--**	x	x	x
C55	Chemnitz 2020	--	--	--	**--**	x	x	x
C56	Chemnitz 2020	--	--	--	**--**	x	x	
C57	Chemnitz 2020	--	--	--	**--**	x	x	
C58	Chemnitz 2020	--	--	--	**--**	x	x	

C59	Chemnitz 2020	--	--	--	--	x	x	
SG70	St. Georg 2020	f	37	--	--	x	x	
L147	LIFE 2015	m	72			x		
L148	LIFE 2015	f	77			x		
L149	LIFE 2015	m	74			x		
L150	LIFE 2015	m	48			x		
L151	LIFE 2015	f	28			x		
L152	LIFE 2015	m	75			x		
L153	LIFE 2015	m	47			x		
L154	LIFE 2015	f	45			x		
L155	LIFE 2015	f	60			x		
L156	LIFE 2015	f	46			x		
L157	LIFE 2015	f	66					x
L158	LIFE 2015	m	47					x
L159	LIFE 2015	f	43					x
L160	LIFE 2015	f	72					x
L161	LIFE 2015	f	45					x
L162	LIFE 2015	f	70					x
L163	LIFE 2015	m	39					x
L164	LIFE 2015	m	32					x
L165	LIFE 2015	f	50					x
L166	LIFE 2015	f	46					x

1. Schwarze, M.; Luo, J.; Brakel, A.; Krizsan, A.; Lakowa, N.; Grünewald, T.; Lehmann, C.; Wolf, J.; Borte, S.; Milkovska-Stamenova, S.; et al. Evaluation of S- and M-Proteins Expressed in Escherichia coli and HEK Cells for Serological Detection of Antibodies in Response to SARS-CoV-2 Infections and mRNA-Based Vaccinations. *Pathogens* **2022**, *11*, doi:10.3390/pathogens11121515

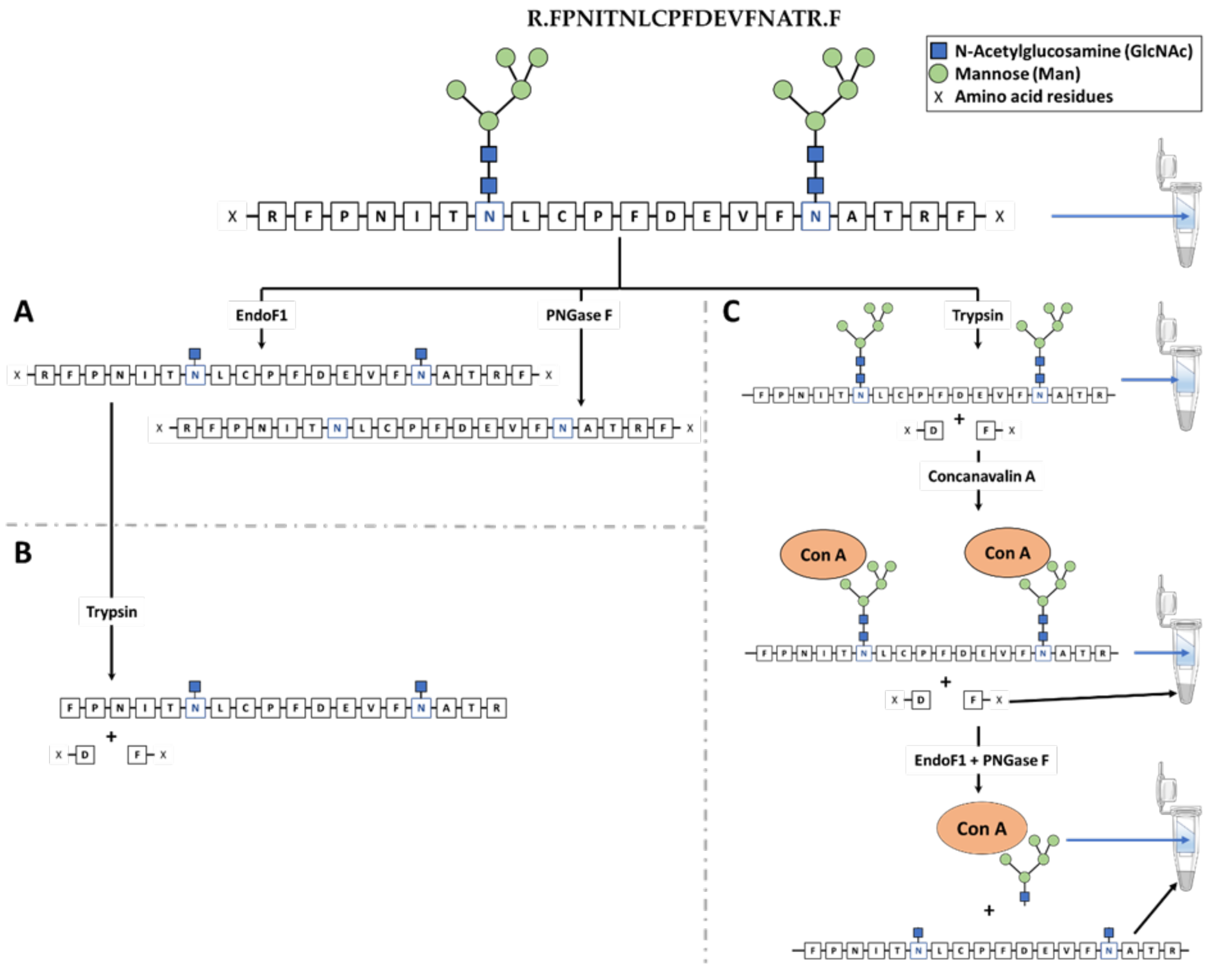

Figure S1. Schematic of the protein preparation protocols applied for SDS-PAGE and ELISA (A) and identification of N-glycosylation sites in RBD proteins using the FASP method (B+C). The first protocol was based on Endo F1 and PNGase F treatments at the protein level followed by tryptic digestion (B), while the second protocol started with a tryptic digestion followed by the addition of the lectin concanavalin A and EndoF1/PNGase F treatment (C) for subsequent mass spectrometric analysis.

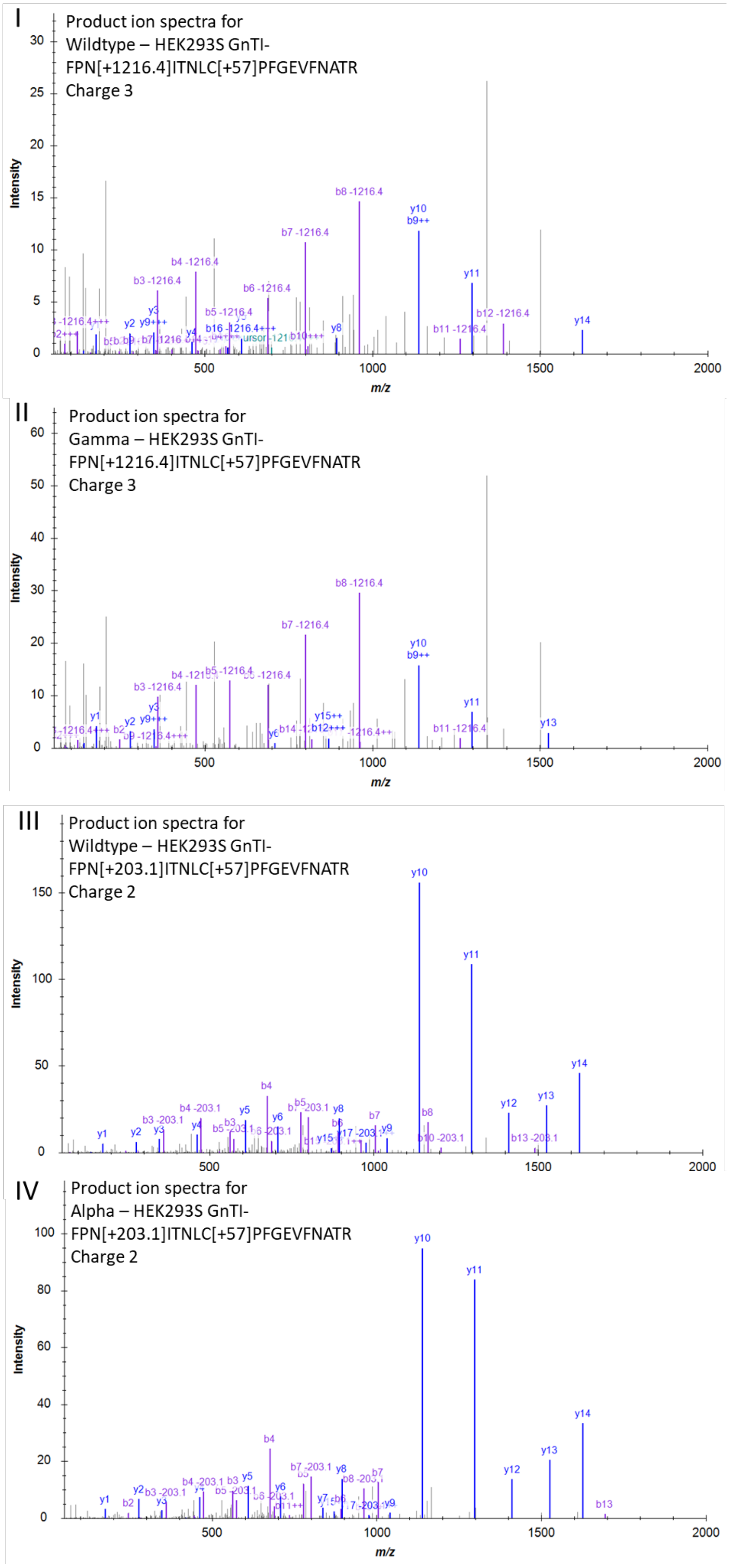
I
Product ion spectra for
Wildtype – HEK293S GnTI-
FPN[+1216.4]ITNLC[+57]PFGEVFNATR
Charge 3
Intensity
m/z
II
Product ion spectra for
Gamma – HEK293S GnTI-
FPN[+1216.4]ITNLC[+57]PFGEVFNATR
Charge 3
Intensity
m/z
III
Product ion spectra for
Wildtype – HEK293S GnTI-
FPN[+203.1]ITNLC[+57]PFGEVFNATR
Charge 2
Intensity
IV
Product ion spectra for
Alpha – HEK293S GnTI-
FPN[+203.1]ITNLC[+57]PFGEVFNATR
Charge 2
Intensity
m/z

V

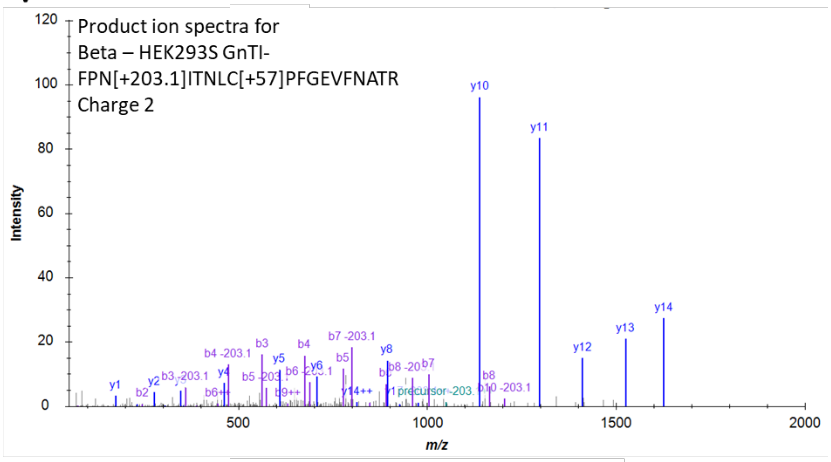

Product ion spectra for
Beta – HEK293S GnTI-
FPN[+203.1]ITNLC[+57]PFGEVFNATR
Charge 2
Intensity
m/z
y10
y11
y12
y13
y14

VI

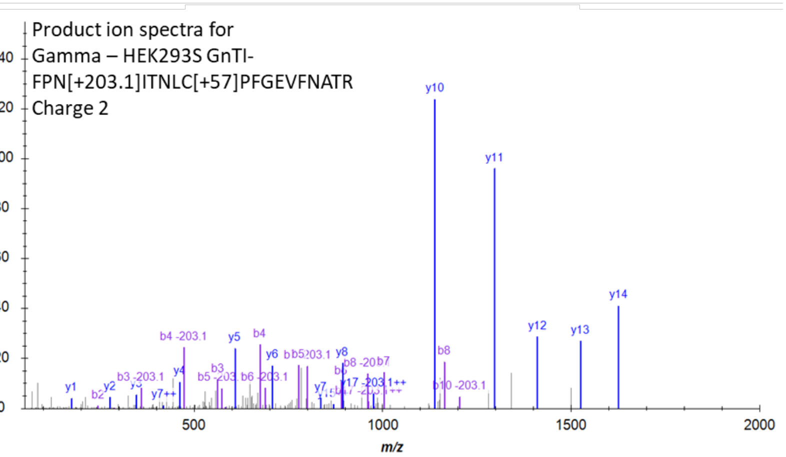

Product ion spectra for
Gamma – HEK293S GnTI-
FPN[+203.1]ITNLC[+57]PFGEVFNATR
Charge 2
Intensity
m/z
y10
y11
y12
y13
y14

VII

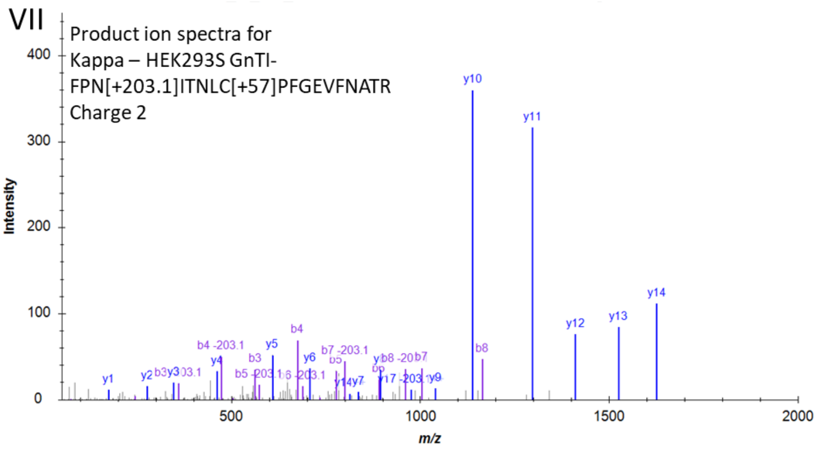

Product ion spectra for
Kappa – HEK293S GnTI-
FPN[+203.1]ITNLC[+57]PFGEVFNATR
Charge 2
Intensity
m/z
y10
y11
y12
y13
y14

VIII

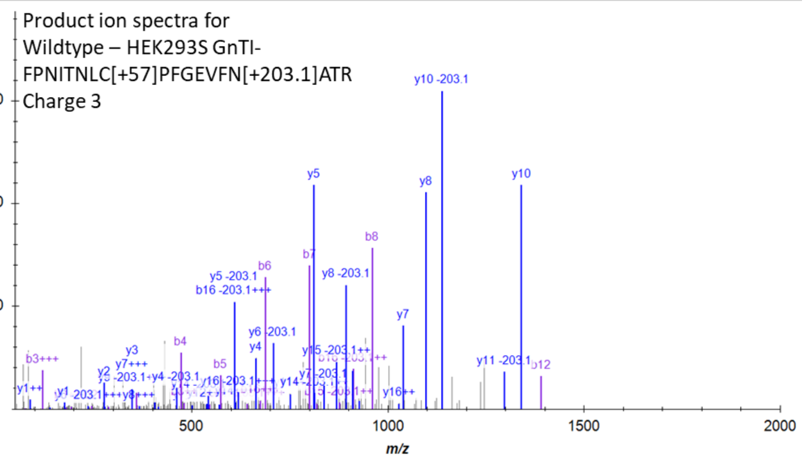

Product ion spectra for
Wildtype – HEK293S GnTI-
FPNITNLC[+57]PFGEVFN[+203.1]ATR
Charge 3
Intensity
m/z
y10 -203.1
y5
y8
y10
b8

IX

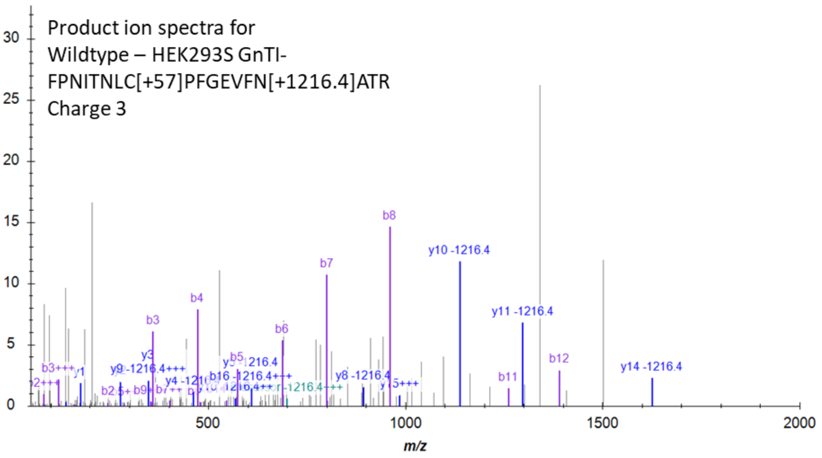
Product ion spectra for
Wildtype – HEK293S GnTI-
FPNITNLC[+57]PFGEVFN[+1216.4]ATR
Charge 3
Intensity
m/z

X

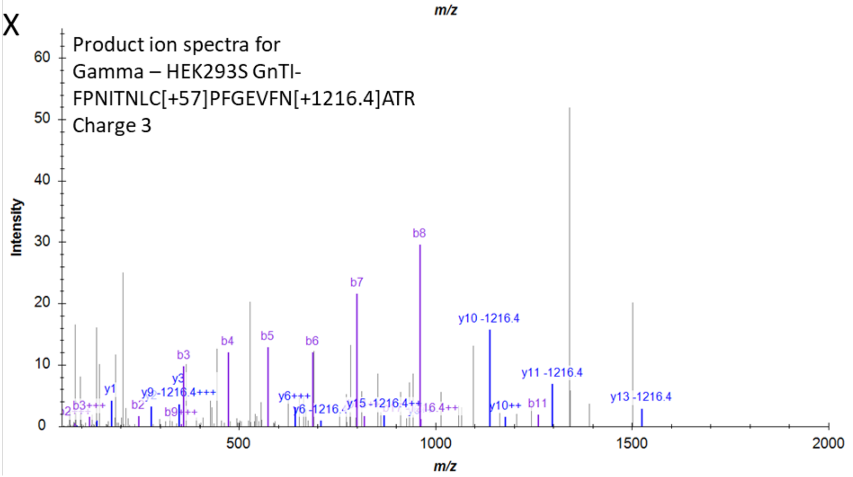
Product ion spectra for
Gamma – HEK293S GnTI-
FPNITNLC[+57]PFGEVFN[+1216.4]ATR
Charge 3
Intensity
m/z

XI

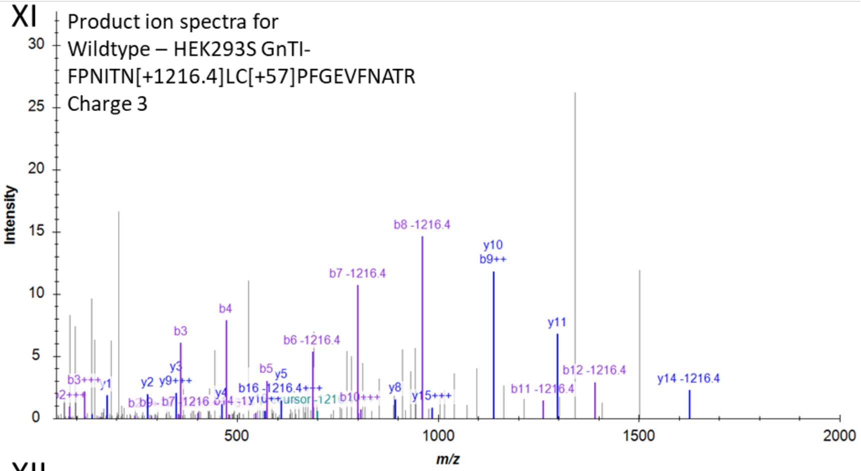
Product ion spectra for
Wildtype – HEK293S GnTI-
FPNITN[+1216.4]LC[+57]PFGEVFNATR
Charge 3
Intensity
m/z

XII

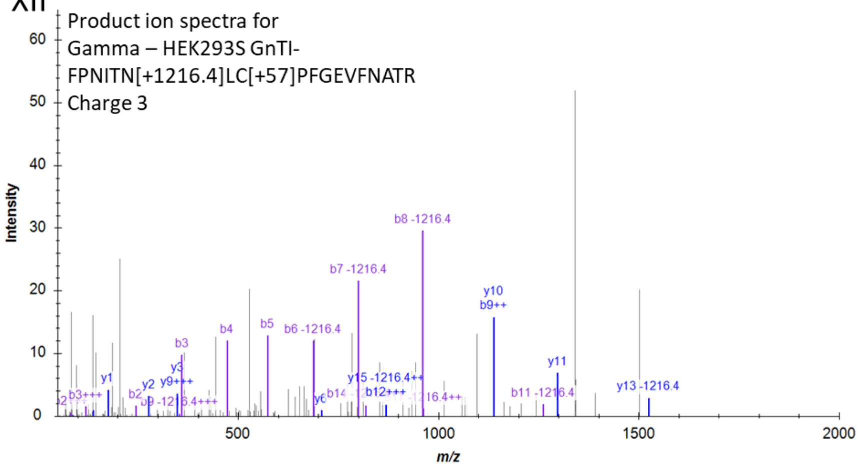
Product ion spectra for
Gamma – HEK293S GnTI-
FPNITN[+1216.4]LC[+57]PFGEVFNATR
Charge 3
Intensity
m/z

XIII

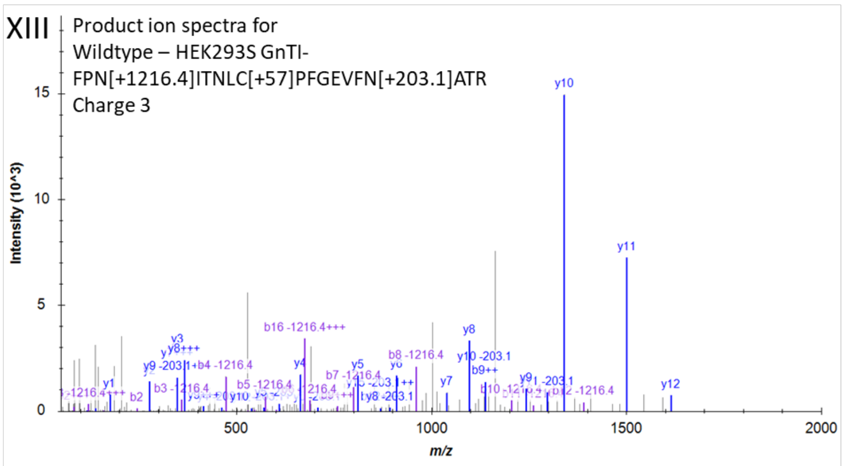

XIV

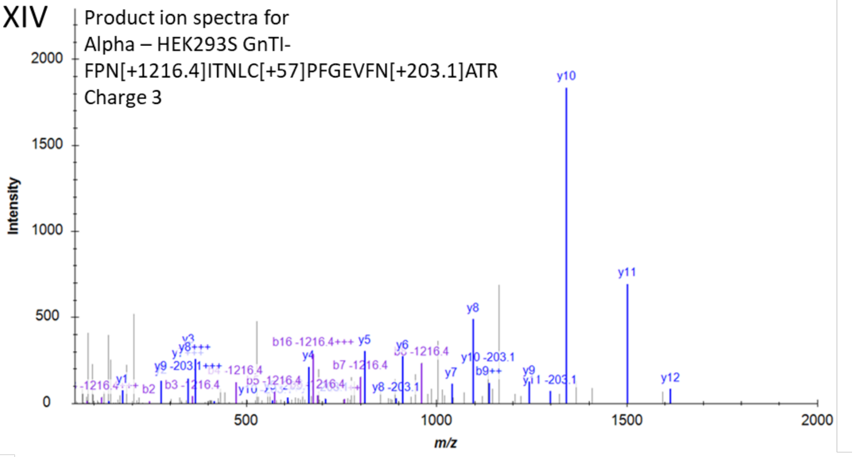

XV

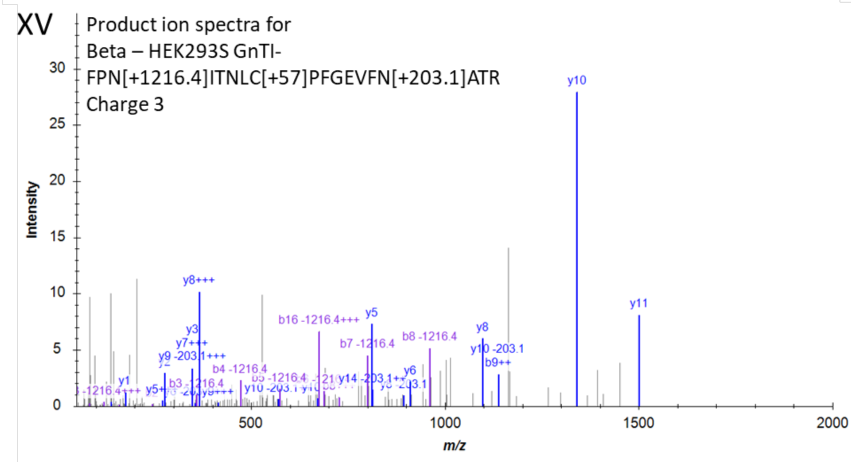

XVI

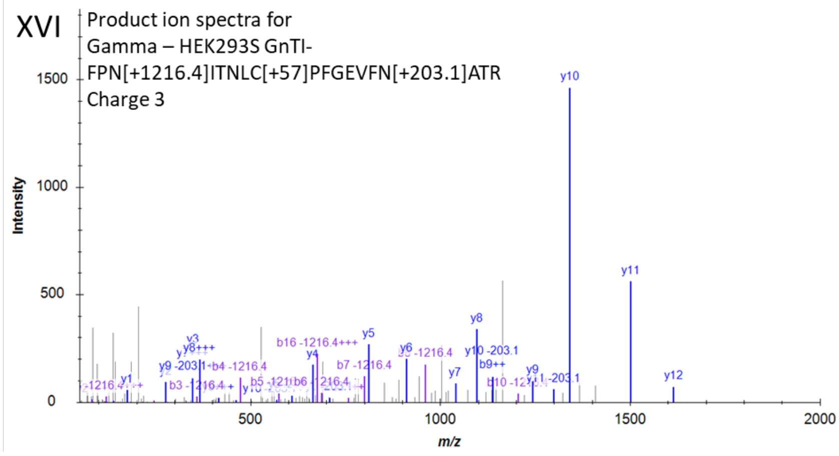

XVII

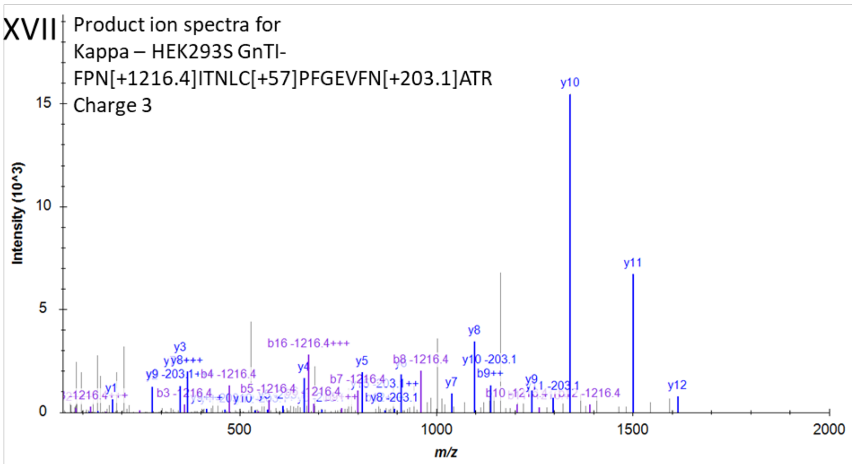

XVIII

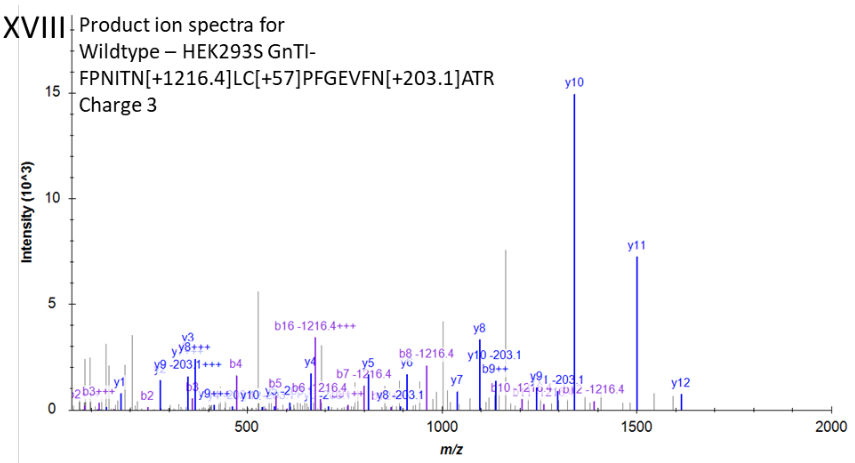

XIX

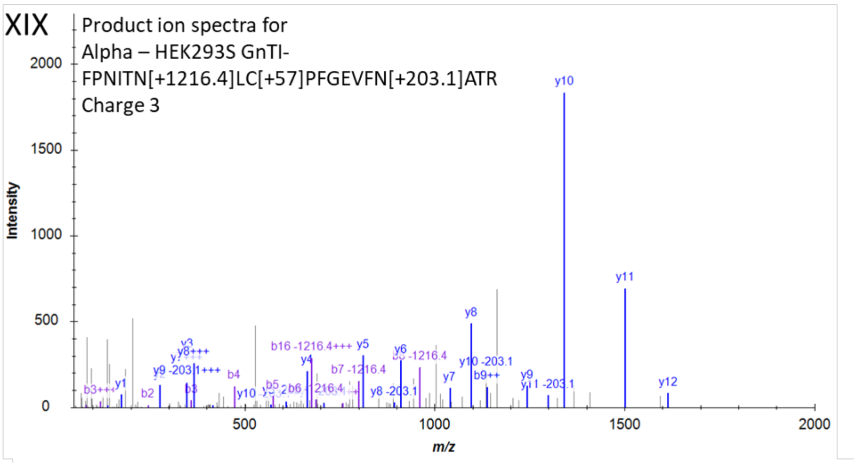

XX

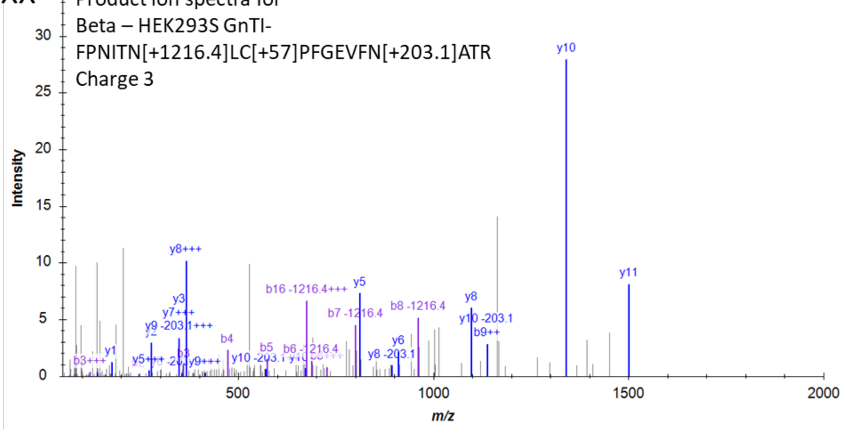

XXI

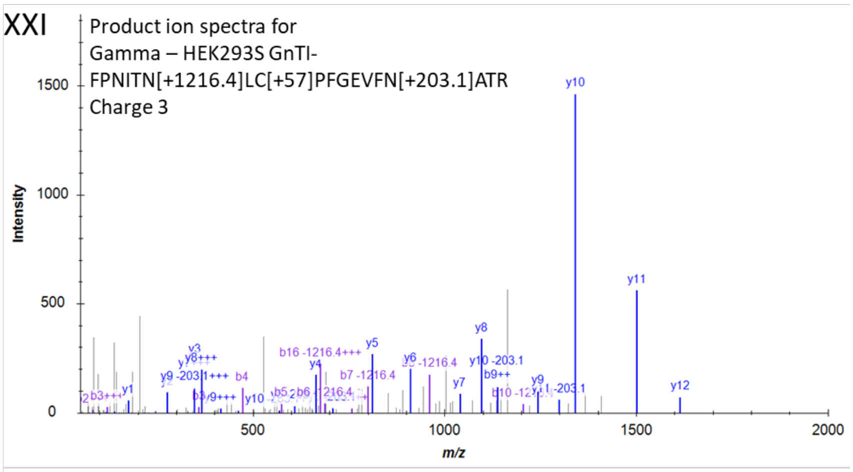

XXII

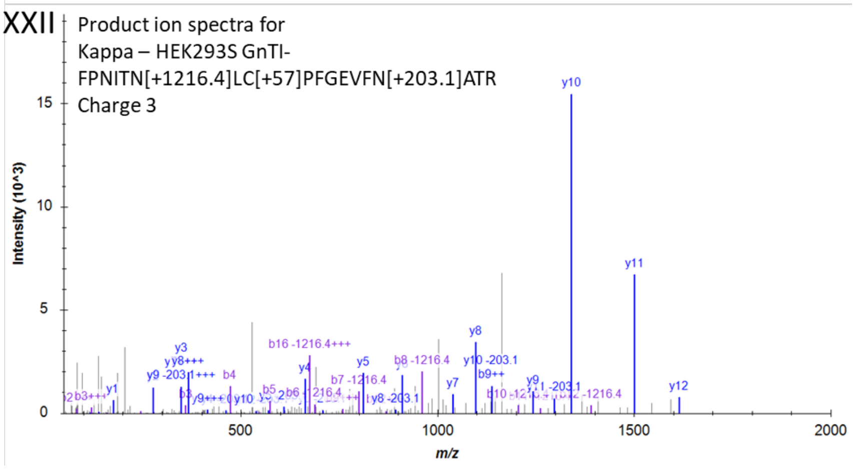

XXIII

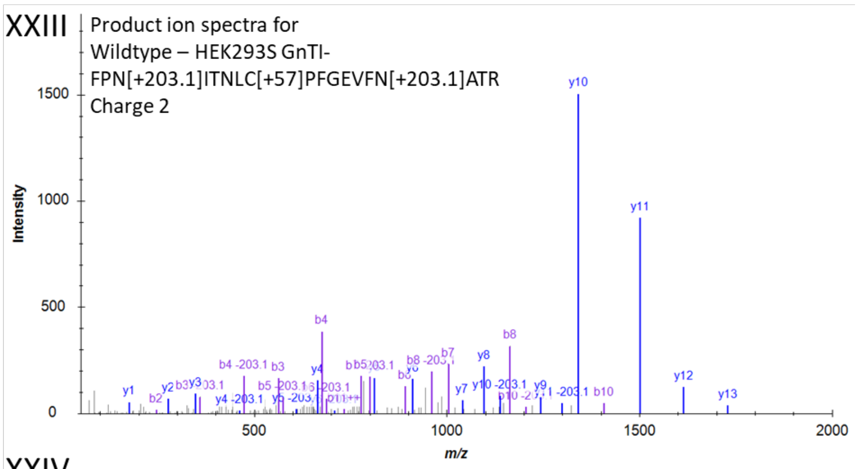

XXIV

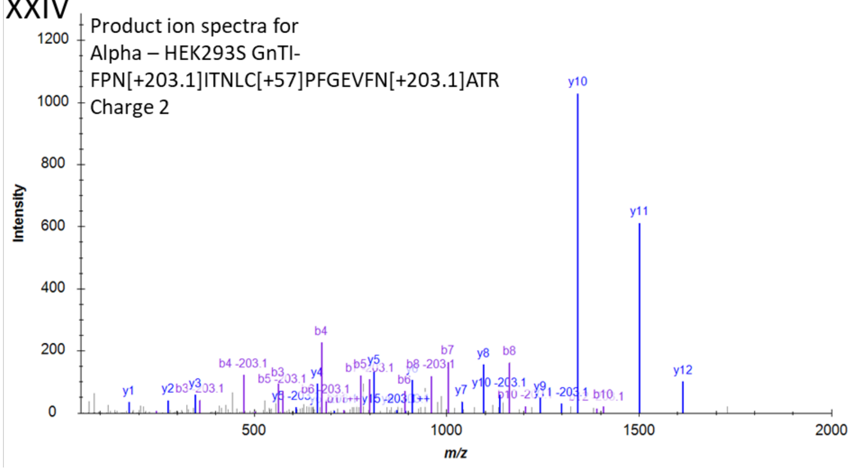

XXV

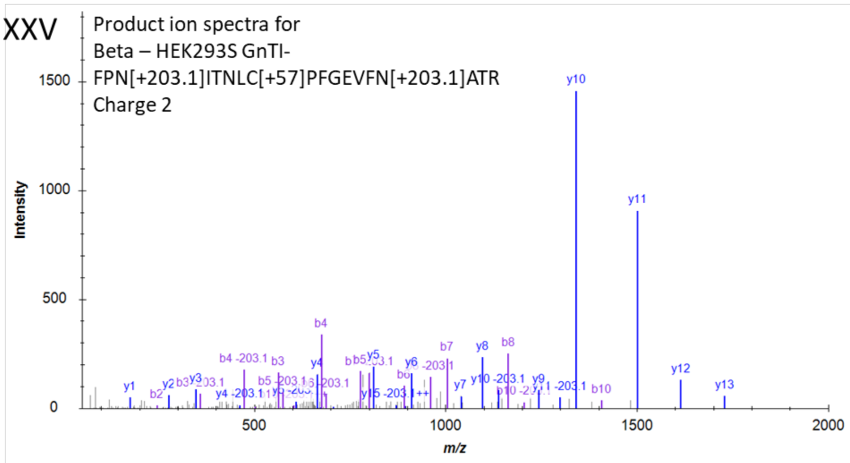

XXVI

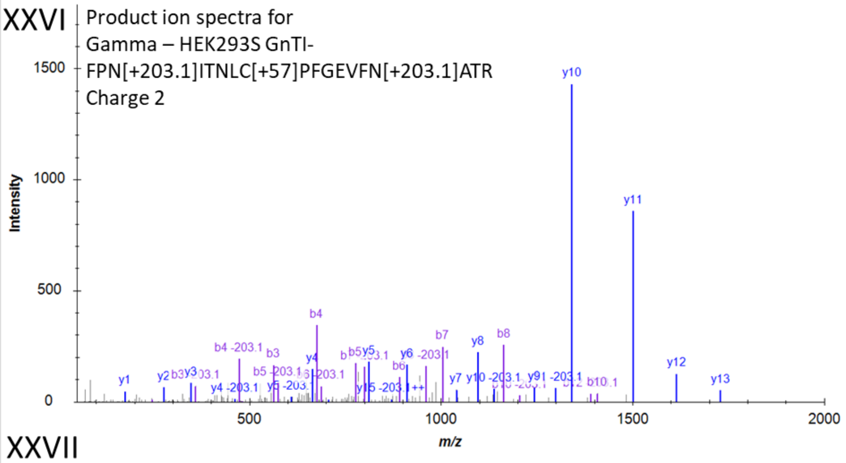

XXVII

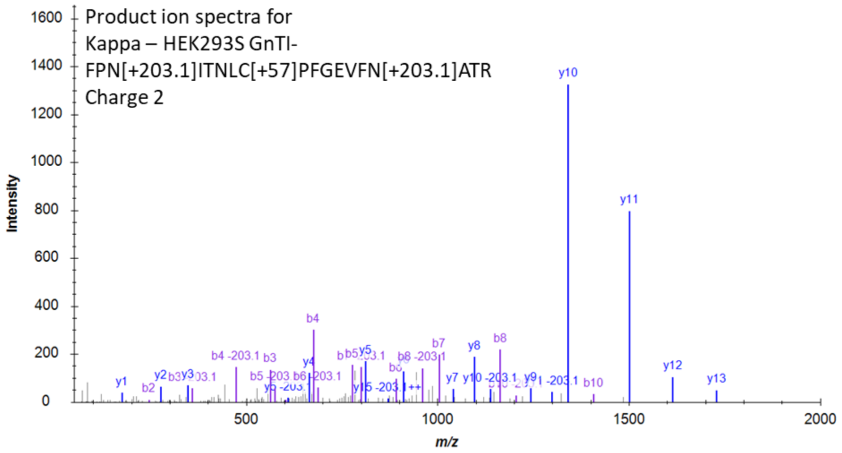

XXVIII

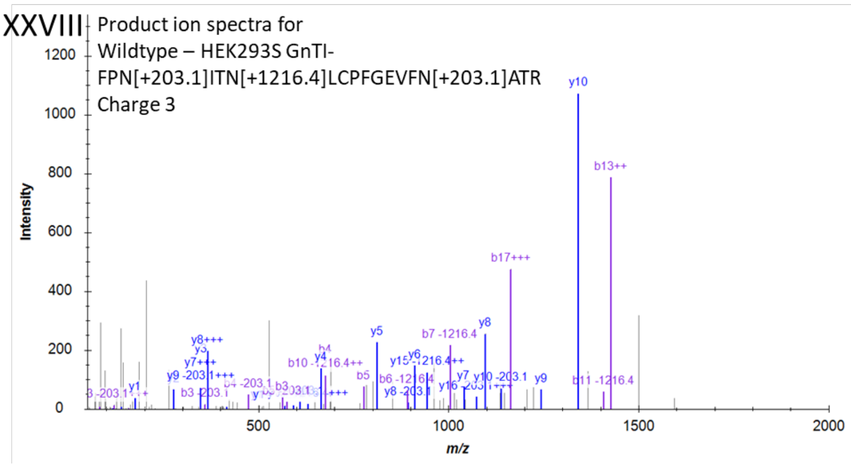

XXIX

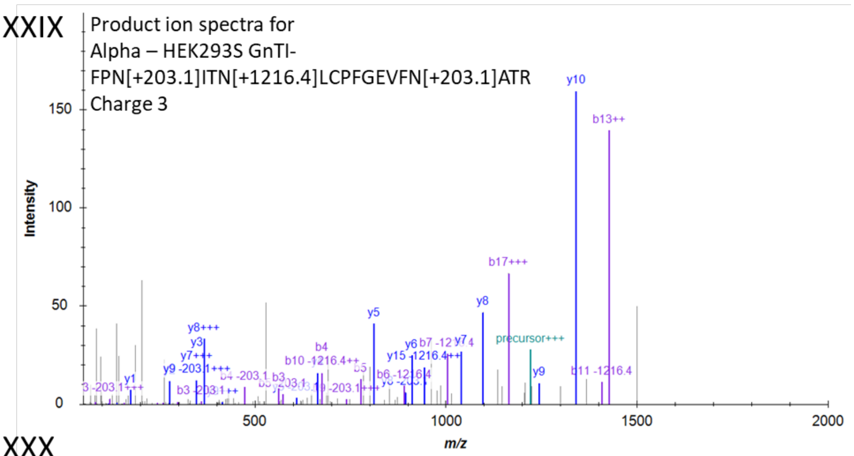

XXX

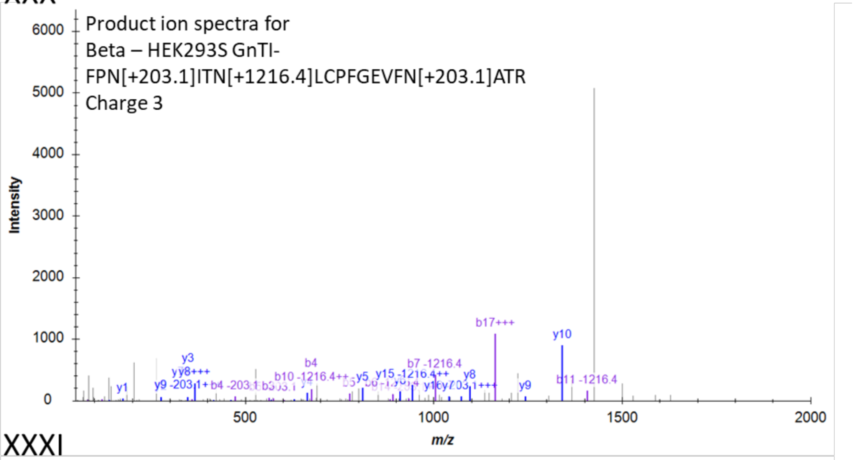

XXXI

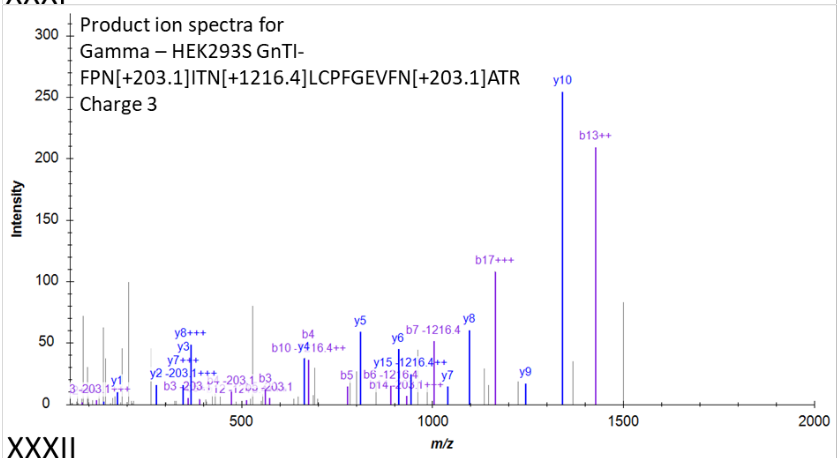

XXXII

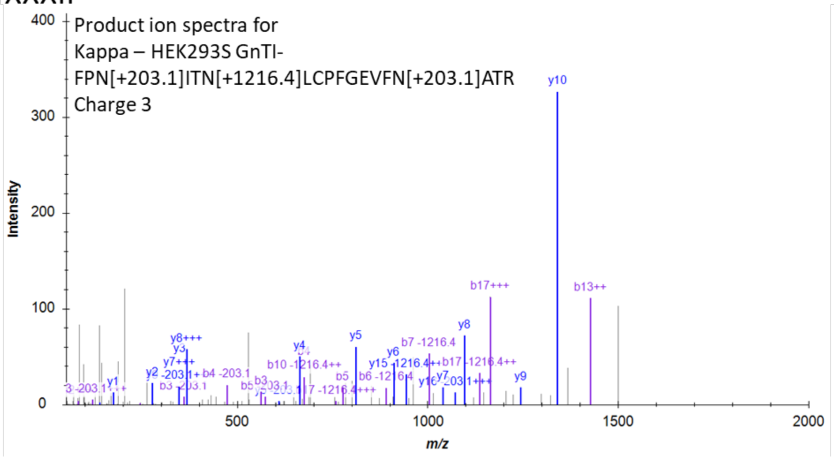

XXXIII

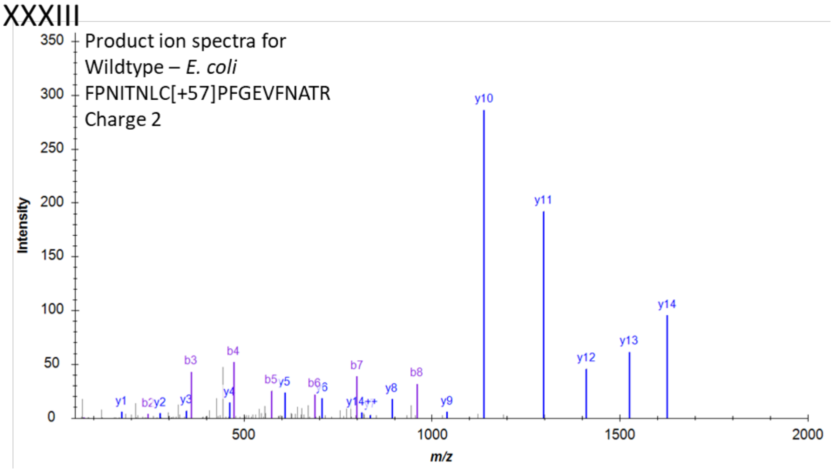

XXXIV

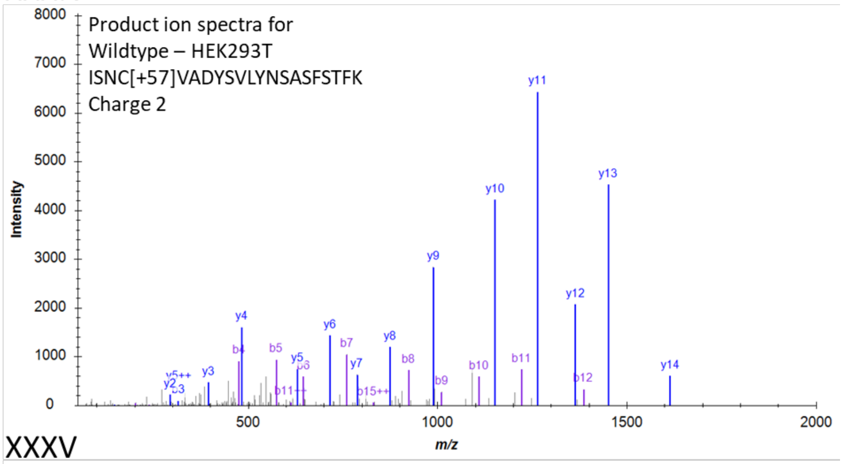

XXXV

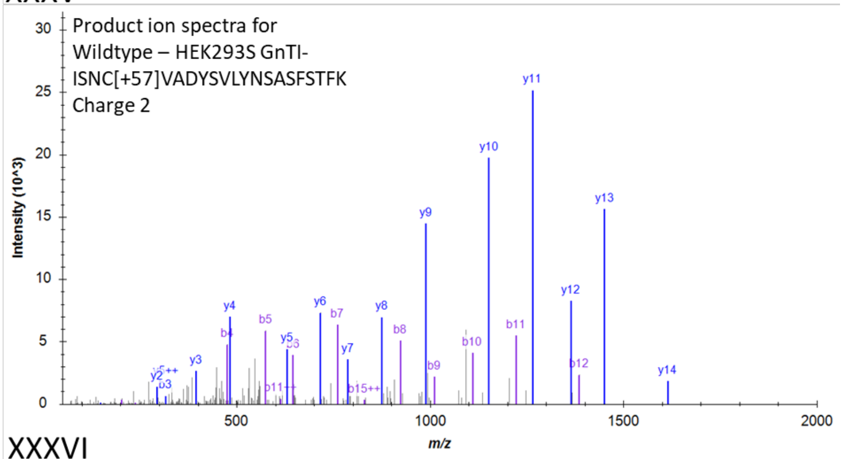

XXXVI

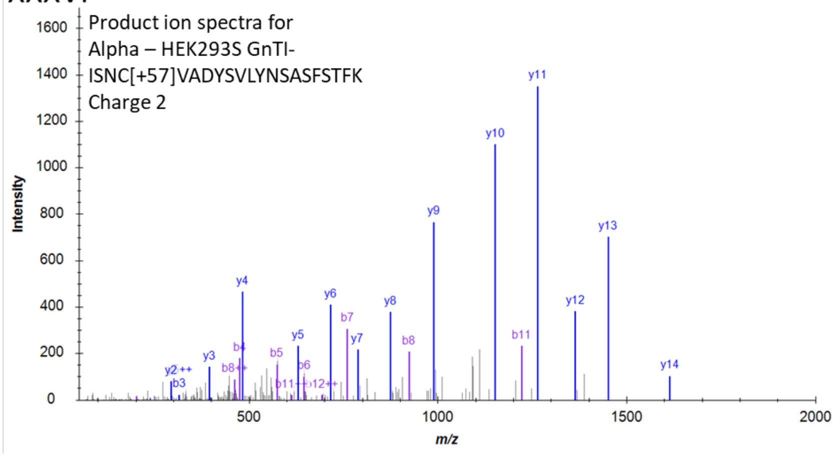

XXXVII

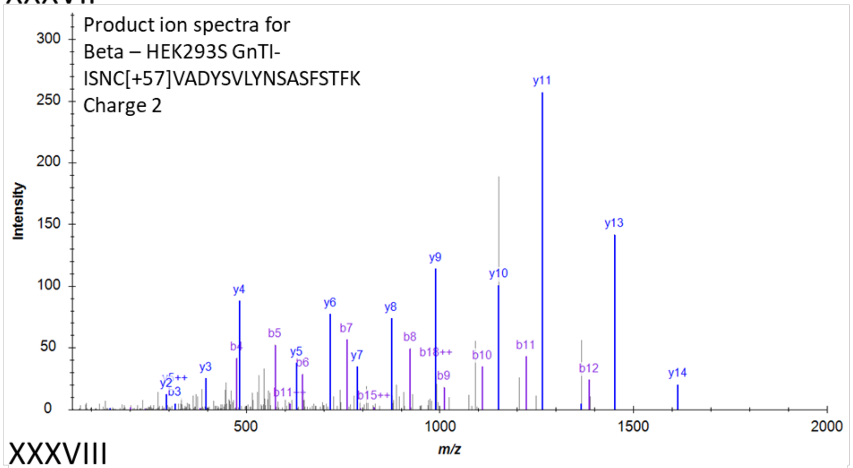

XXXVIII

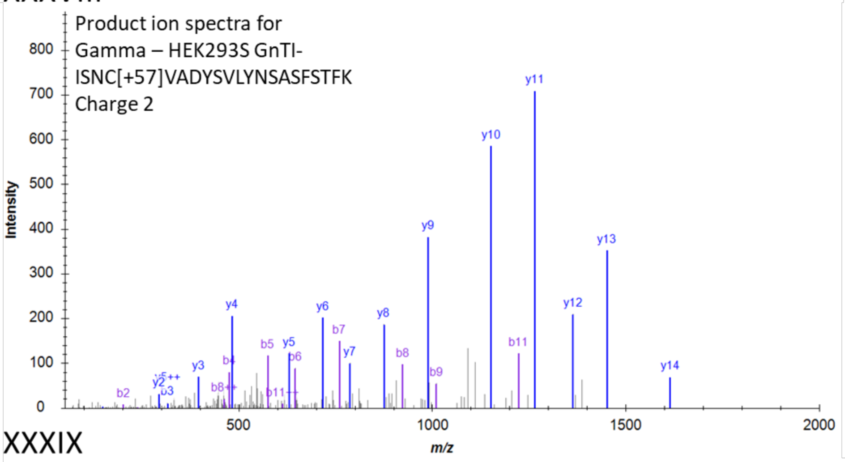

XXXIX

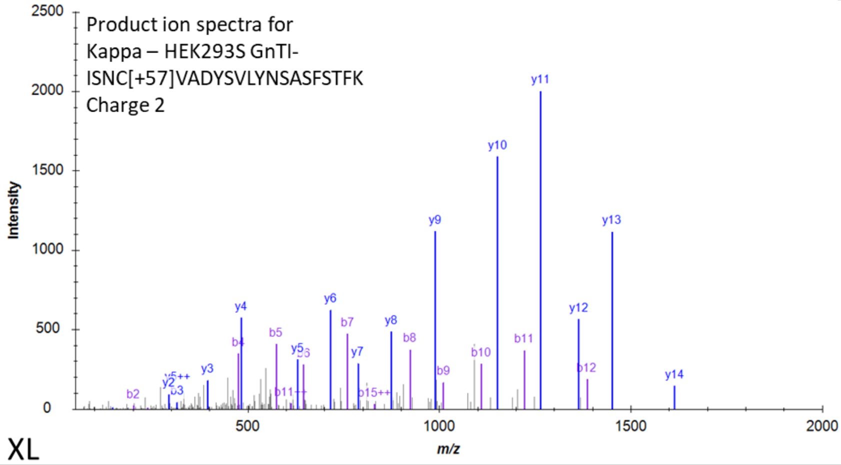

XL

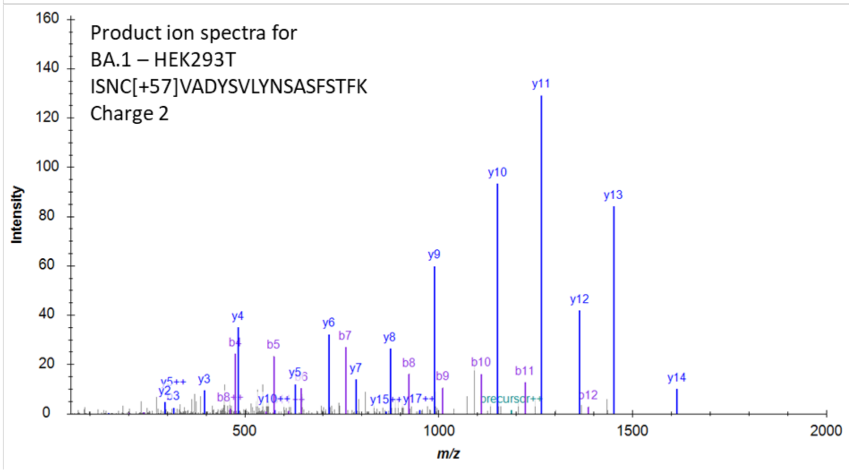

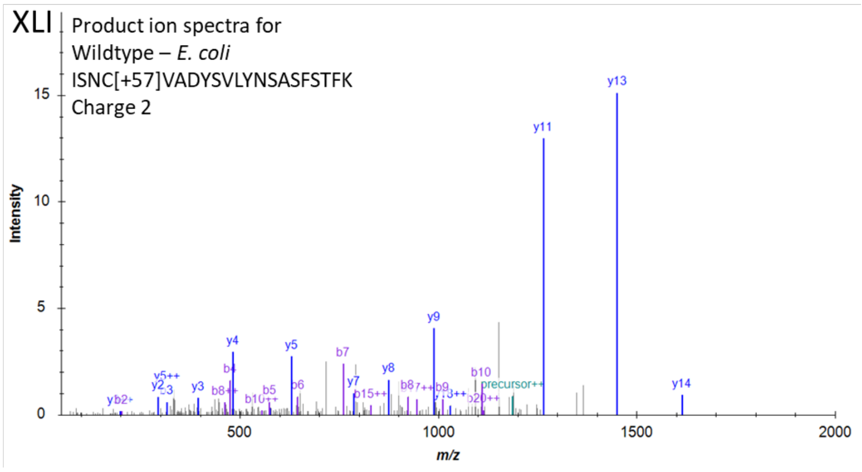
XLI
Product ion spectra for
Wildtype – *E. coli*
ISNC[+57]VADYSVLYNSASFSTFK
Charge 2
Intensity
m/z
y13
y11
y9
y4
y5
b7
y8
b10
precursor++
y14

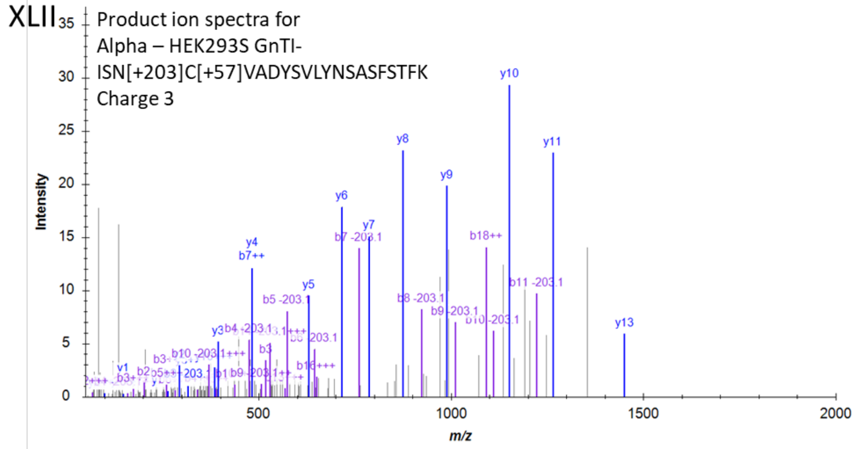
XLII
Product ion spectra for
Alpha – HEK293S GnTI-
ISN[+203]C[+57]VADYSVLYNSASFSTFK
Charge 3
Intensity
m/z
y10
y8
y11
y9
y6
y7
b18++
y4
b7++
y5
y13

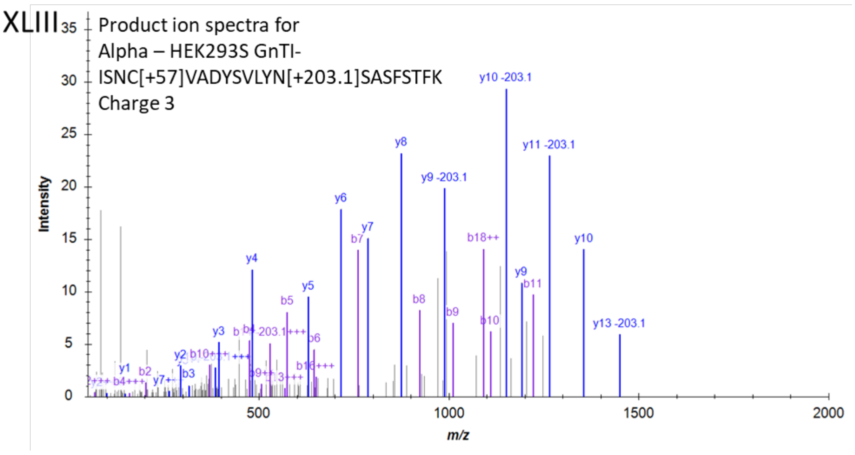
XLIII
Product ion spectra for
Alpha – HEK293S GnTI-
ISNC[+57]VADYSVLYN[+203.1]SASFSTFK
Charge 3
Intensity
m/z
y10 -203.1
y8
y11 -203.1
y9 -203.1
y6
y7
b18++
y10
y4
y5
y13 -203.1

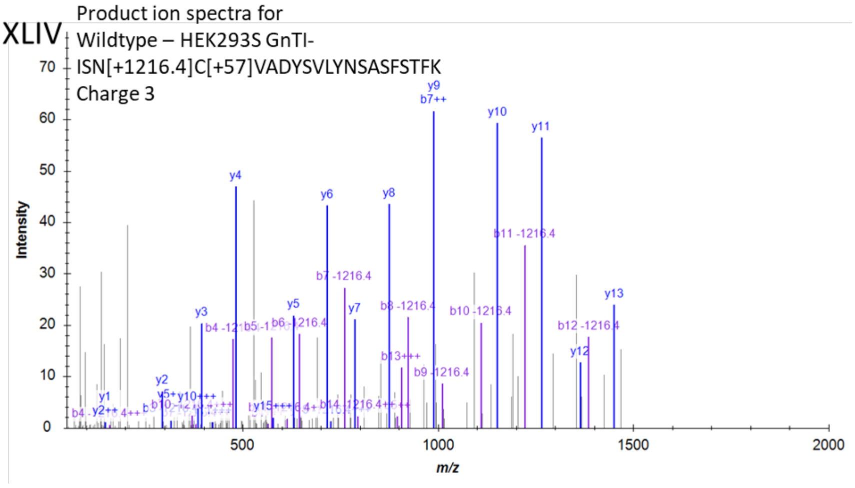
XLIV
Product ion spectra for
Wildtype – HEK293S GnTI-
ISN[+1216.4]C[+57]VADYSVLYNSASFSTFK
Charge 3
Intensity
m/z
y9
b7++
y10
y11
y4
y6
y8
b11 -1216.4
b7 -1216.4
y13
y5
y3
y7
y12

XLV

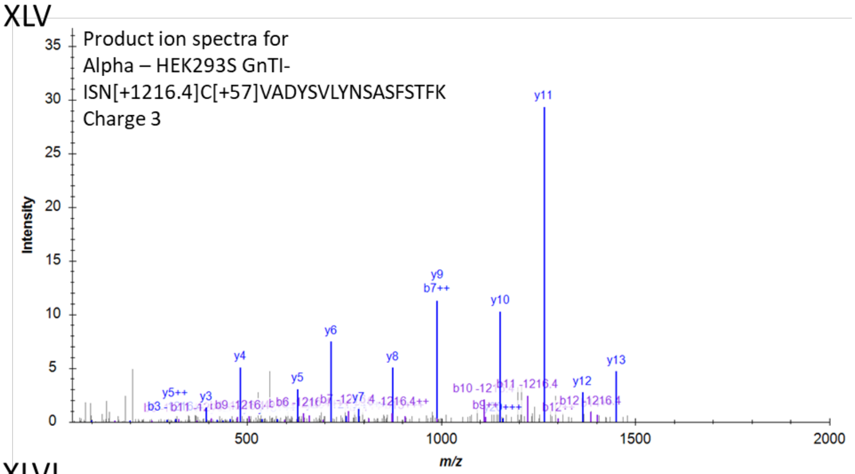

XLVI

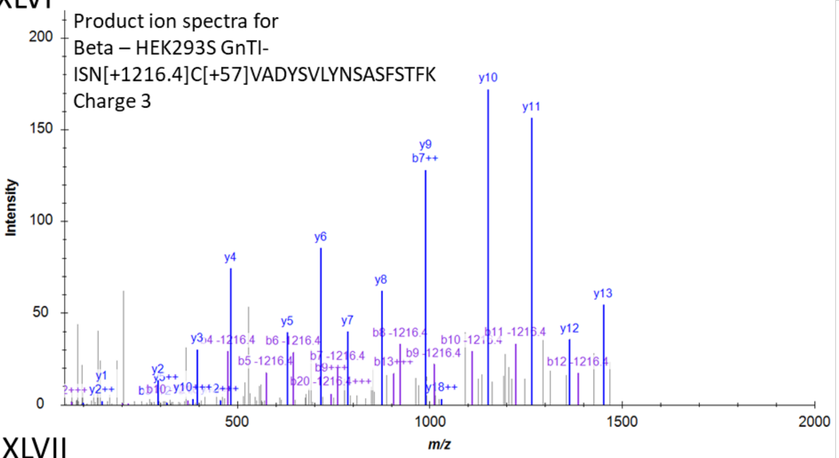

XLVII

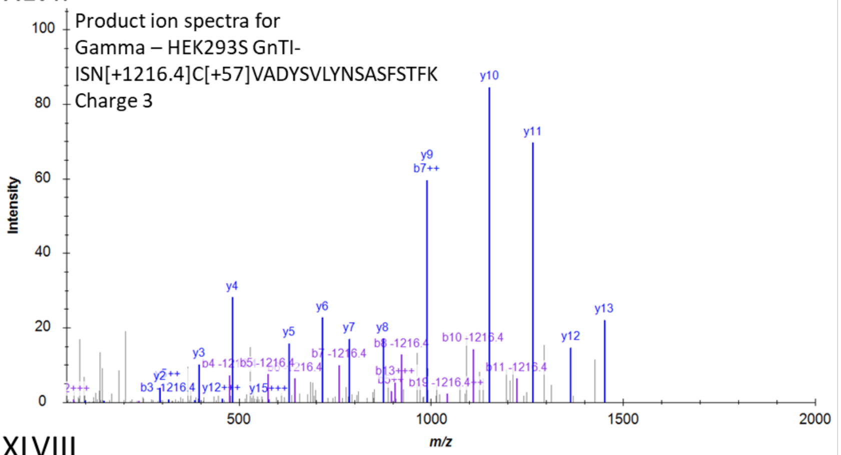

XLVIII

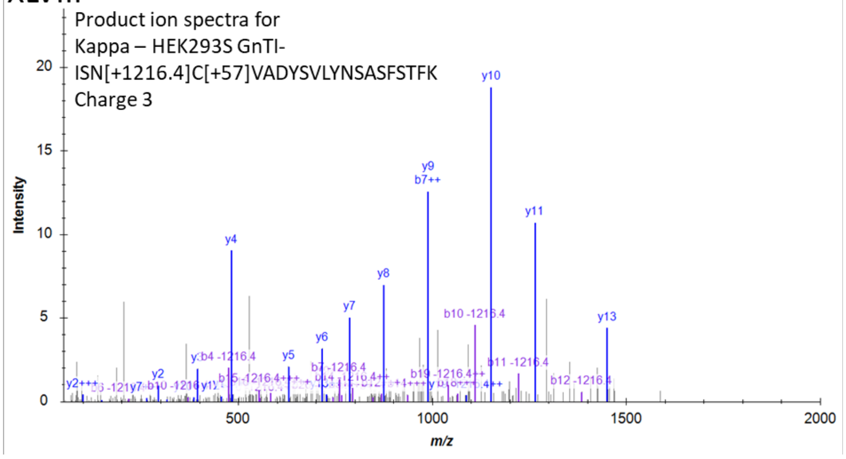

XLIX

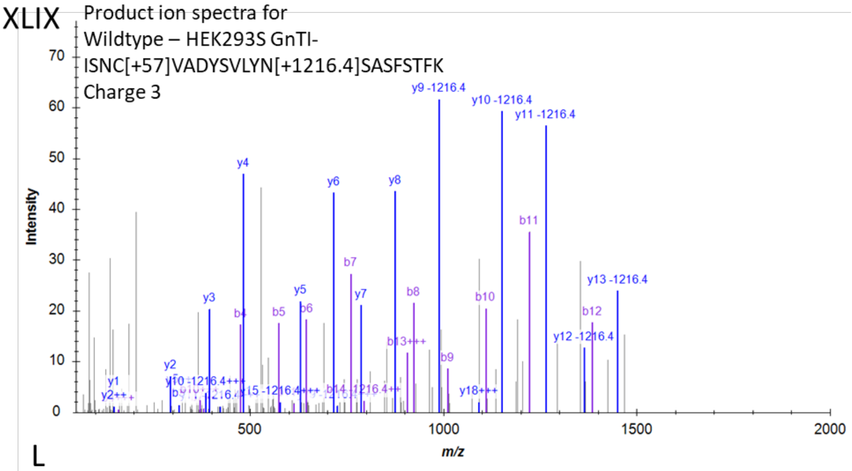
Product ion spectra for
Wildtype – HEK293S GnTI-
ISNC[+57]VADYSVLYN[+1216.4]SASFSTFK
Charge 3
Intensity
m/z

L

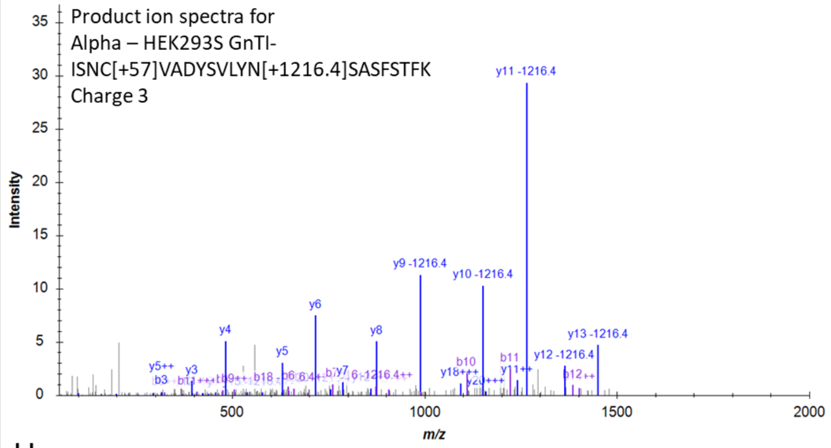
Product ion spectra for
Alpha – HEK293S GnTI-
ISNC[+57]VADYSVLYN[+1216.4]SASFSTFK
Charge 3
Intensity
m/z

LI

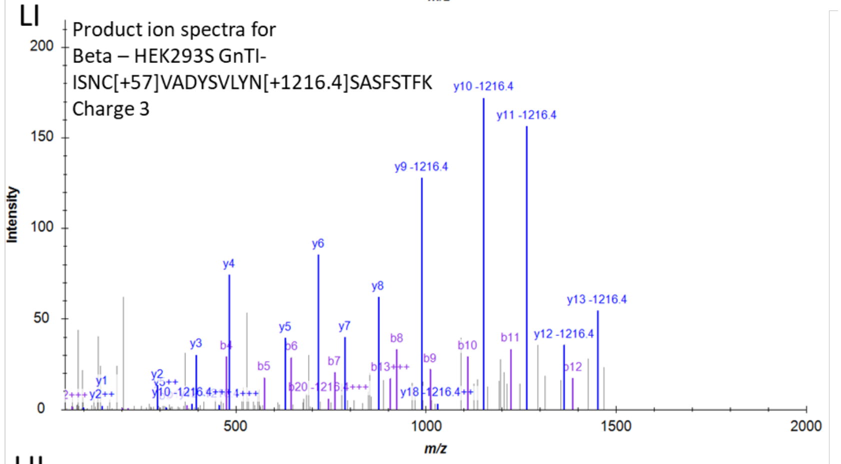
Product ion spectra for
Beta – HEK293S GnTI-
ISNC[+57]VADYSVLYN[+1216.4]SASFSTFK
Charge 3
Intensity
m/z

LII

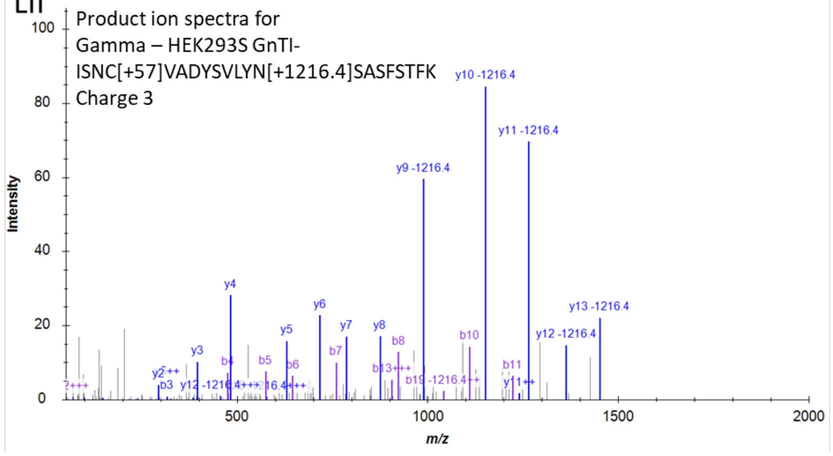
Product ion spectra for
Gamma – HEK293S GnTI-
ISNC[+57]VADYSVLYN[+1216.4]SASFSTFK
Charge 3
Intensity
m/z

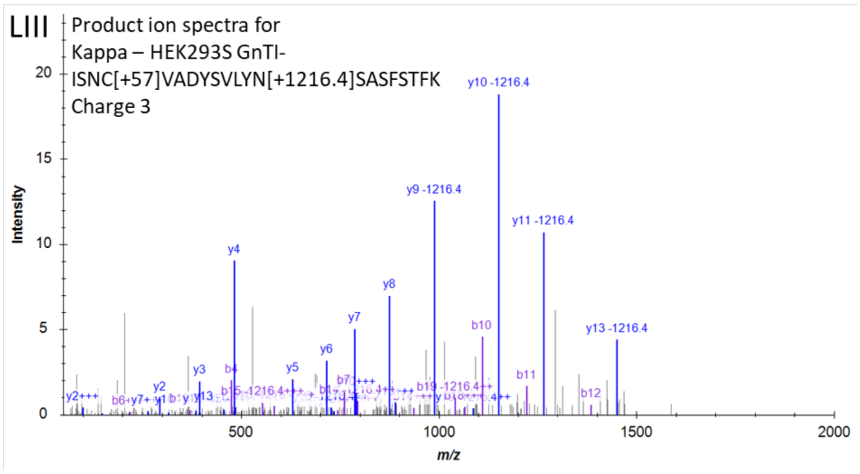

Figure S2. Product ion spectra acquired for tryptic digests of Endo F1-treated wildtype and VOC-RBDs annotated as glycosylated at asparagine residues of peptides FPNITNLCPFGEVFNATR (panels I to XXXIII) and ISNCVADYSVLYNSASFSTFK (XXXIV to LIII). The mass spectra shown in panels I, III, VIII, IX, XI, XIII, XVIII, XXIII, XXVIII, XXXV, XLIV, and XLIX were obtained from the tryptic digests of Endo F1-treated wildtype RBD expressed in HEK293S GnTI- cells. The mass spectrum shown in panel XXXIV was obtained from the tryptic digest of Endo F1-treated RBD expressed in HEK293T cells. The mass spectra shown in panels XXXIII and XLI were obtained from the tryptic digests of RBD expressed in *E. coli*. The mass spectra shown in panels IV, XIV, XIX, XXIV, XXIX, XXXVI, XLII, XLIII, XLV, and L were obtained from the tryptic digests of Endo F1-treated alpha RBD expressed in HEK293S GnTI- cells. The mass spectra shown in panels V, XV, XX, XXV, XXX, XXXVII, XLVI, and LI were obtained from the tryptic digests of Endo F1-treated beta RBD expressed in HEK293S GnTI- cells. The mass spectra shown in panels II, VI, X, XII, XVI, XXI, XXVI, XXXI, XXXVIII, XLVII, and LII were obtained from the tryptic digests of Endo F1-treated gamma RBD expressed in HEK293S GnTI- cells. The mass spectra shown in panels VII, XVII, XXII, XXVII, XXXII, XXXIX, XLVIII, and LIII were obtained from the tryptic digests of Endo F1-treated kappa RBD expressed in HEK293S GnTI- cells. The mass spectrum shown in panel XL was obtained from the tryptic digest of Endo F1-treated BA.1 RBD expressed in HEK293T cells. Annotated y- and b-ions are colored in blue and purple, respectively, and the precursor ions are colored in light blue.

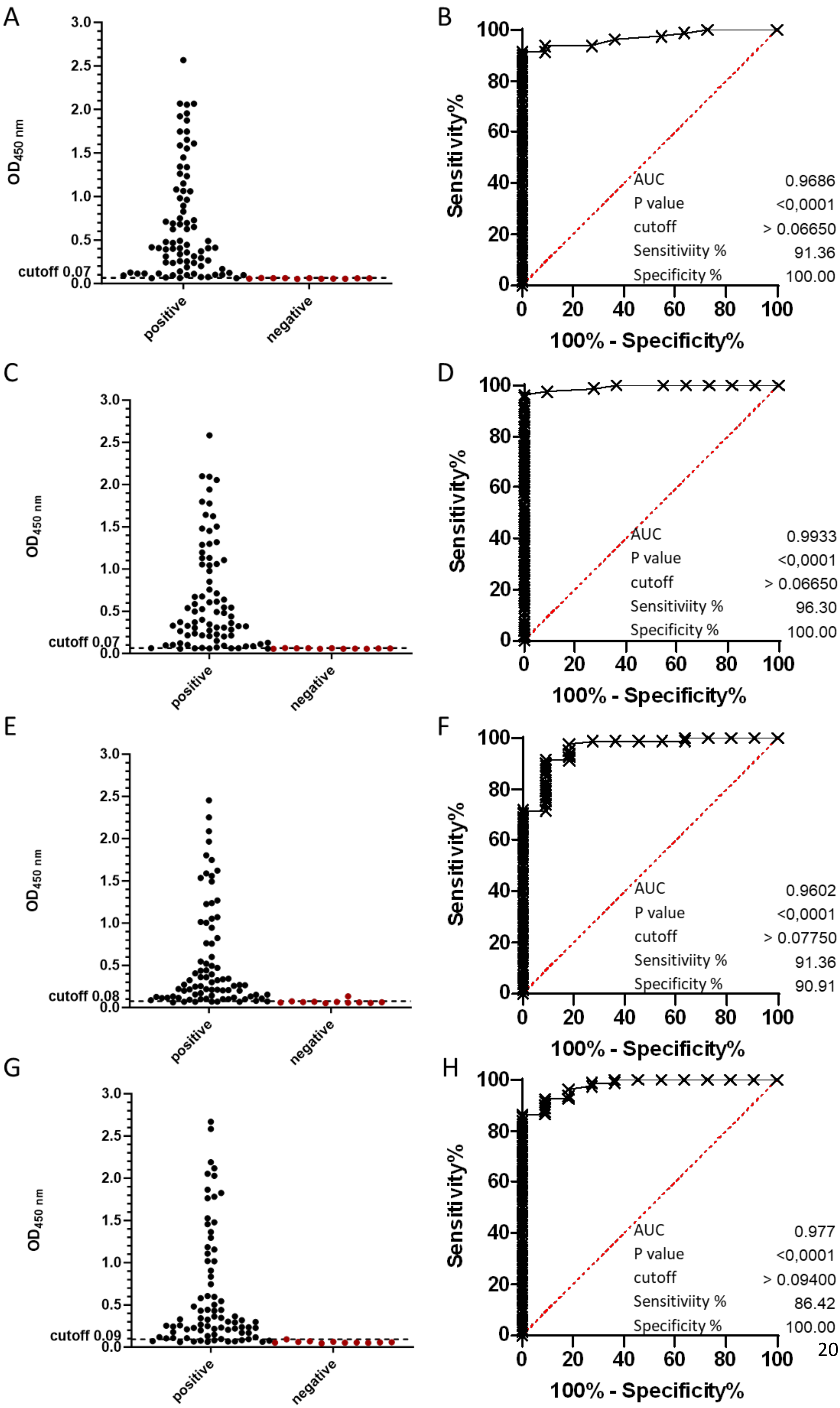
A
B
C
D
E
F
G
H
OD450 nm
cutoff 0.07
cutoff 0.08
cutoff 0.09
positive
negative
Sensitivity%
100% - Specificity%
AUC 0.9686
P value <0,0001
cutoff > 0.06650
Sensitiviity % 91.36
Specificity % 100.00
AUC 0.9933
P value <0,0001
cutoff > 0.06650
Sensitiviity % 96.30
Specificity % 100.00
AUC 0.9602
P value <0,0001
cutoff > 0.07750
Sensitiviity % 91.36
Specificity % 90.91
AUC 0.977
P value <0,0001
cutoff > 0.09400
Sensitiviity % 86.42
Specificity % 100.00

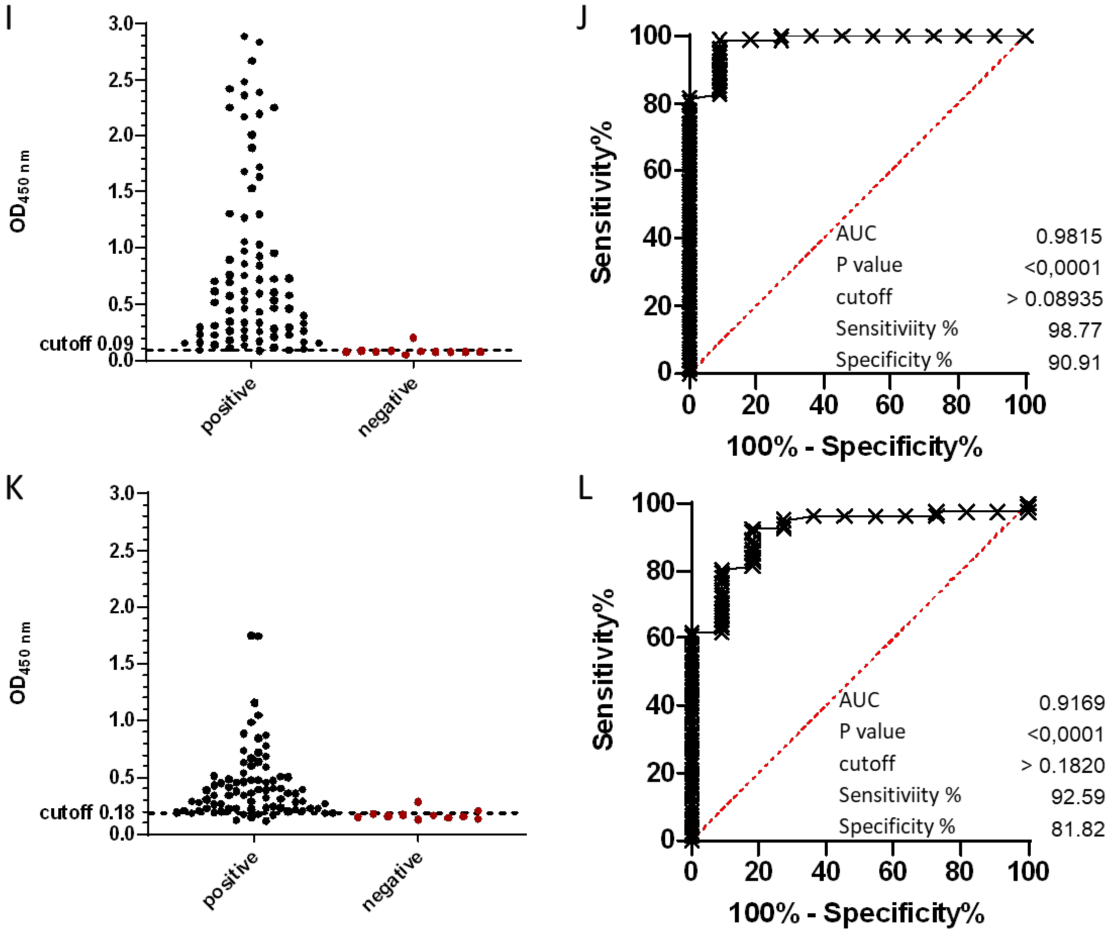

Figure S3. OD_{450} values (left) and calculated ROC curves (right) of the IgG ELISA using different VOC RBDs in a sample set of 81 SARS-CoV-2 positive (black) and 10 negative control samples (red) including also positive and negative pools, created with GraphPad Prism 10.0.3. The cutoff was determined using the Youden index. A, B: wt-RBD, C, D: alpha-RBD; E, F: beta-RBD; G, H: gamma-RBD; I, J: kappa-RBD; K, L: BA.1-RBD.

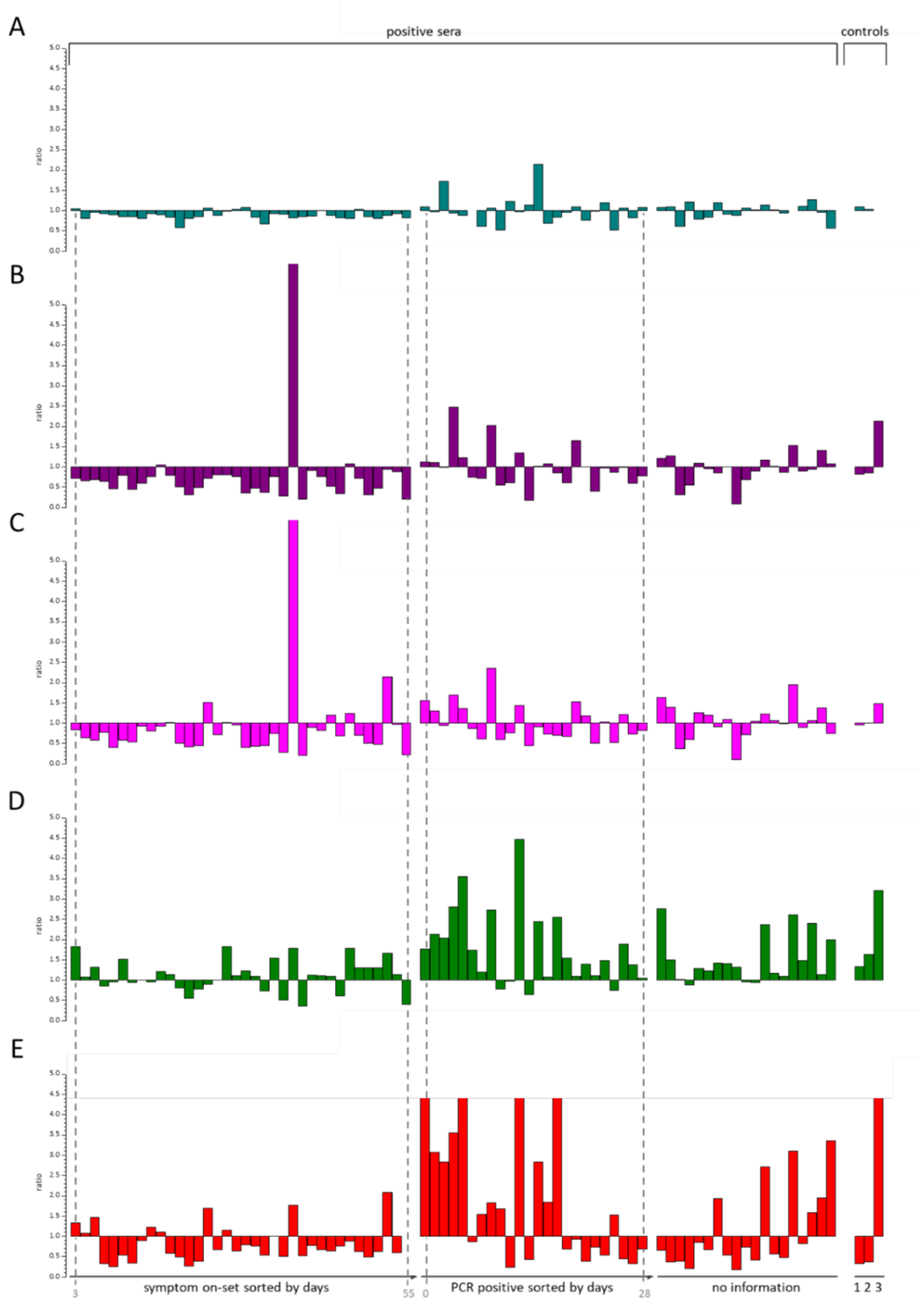

Figure S4. Normalized OD_{450} values obtained for all serum samples using the IgG ELISA coated with different VOC RBDs. OD_{450} values were normalized to the wildtype HEK293S RBD: OD_{450}[X-RBD]/OD_{450}[wt-RBD]; A: Alpha (cyan), B: Beta (purple), C: Gamma (magenta), D: Kappa (green) and E: BA.1 (red). Ratios greater than 1 and less than 1 indicate better and weaker binding, respectively, compared to wt-RBD. Serum samples are divided into four segments. Segment 1: Samples with symptom onset information sorted by ascending days. Segment 2: PCR positive samples in ascending day order. Segment 3: Positive samples with no further information. Segment 4: Control samples prepared by pooling positive (1, 2), and negative serum samples (3). The values were used to calculate the mean values shown in Figure 3.

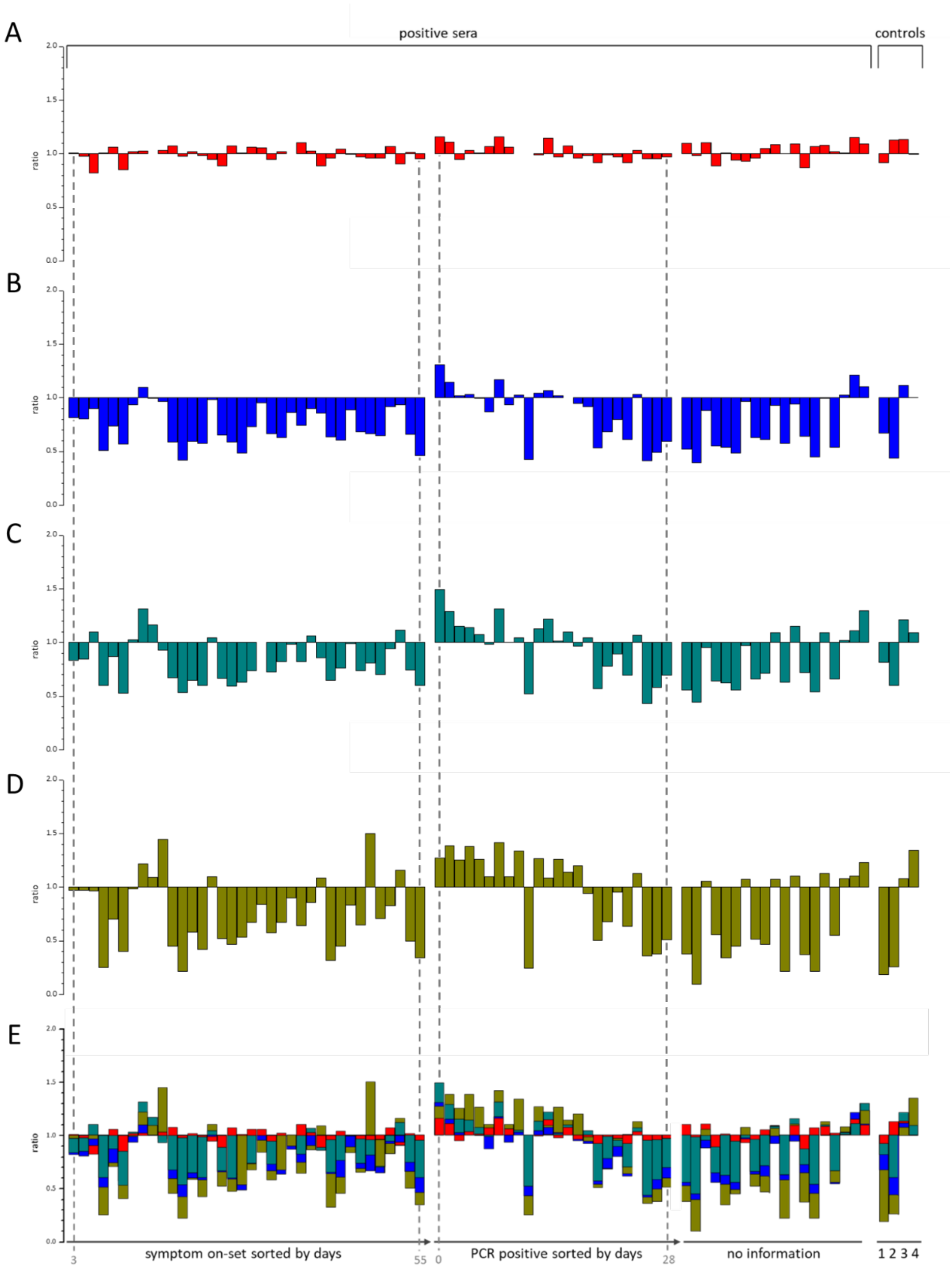

Figure S5. Normalized OD_{450} values obtained for all serum samples using the IgG ELISA coated with different glycosylated RBDs. OD_{450} values were normalized to the wild-type HEK293S RBD: OD_{450}[X-RBD]/OD_{450}[S-RBD]; A: T-RBD (red), B: F1-RBD (blue), C: PNG-RBD (cyan), D: *E. coli* RBD (green), E: RBDs from panels A to D combined. Ratios greater than 1 and less than 1 indicate better and weaker binding, respectively, compared to S-RBD. Serum samples are divided into four segments. Segment 1: Samples with symptom onset information sorted by ascending days. Segment 2: PCR positive samples in ascending day order. Segment 3: Positive samples with no further information. Segment 4: Control samples prepared by pooling high positive (1), positive (2), and negative serum samples (3 for pool I, 4 for pool II). The values were used to calculate the mean values shown in Figure 4.

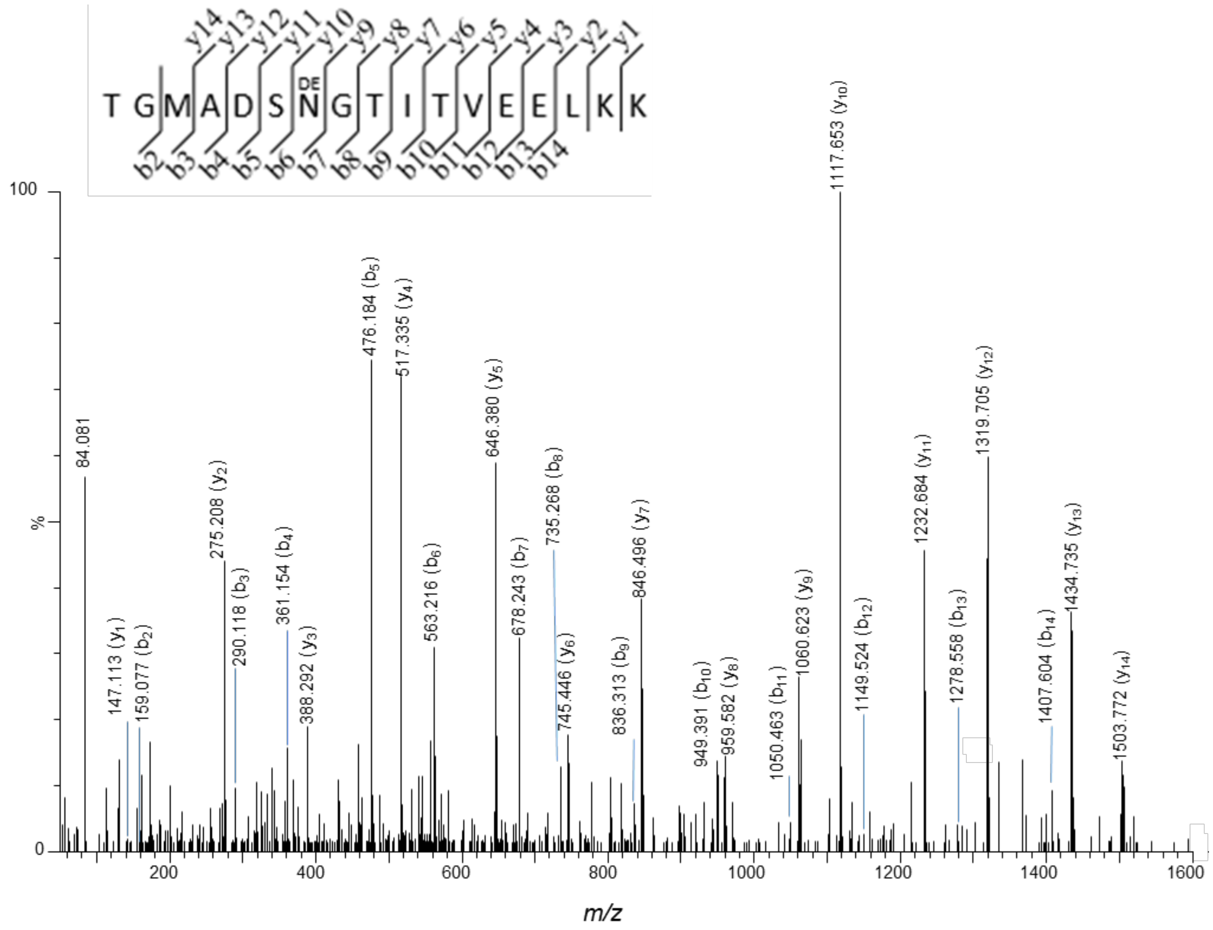

Figure S6. Product ion spectrum acquired for a peptide detected in the tryptic digest of the membrane protein as a doubly protonated ion at *m/z* 897.949. The M-protein was expressed in HEK293S GnTI- cells, digested with trypsin, and enriched with concanavallin A before incubation with Endo F1 and PNGase F. The annotated b- and y-ions suggested the sequence TGMADSN[+1]GTITVEELKK, with the mass shift of +1 at asparagine indicating an aspartic acid residue due to PNGase F treatment.

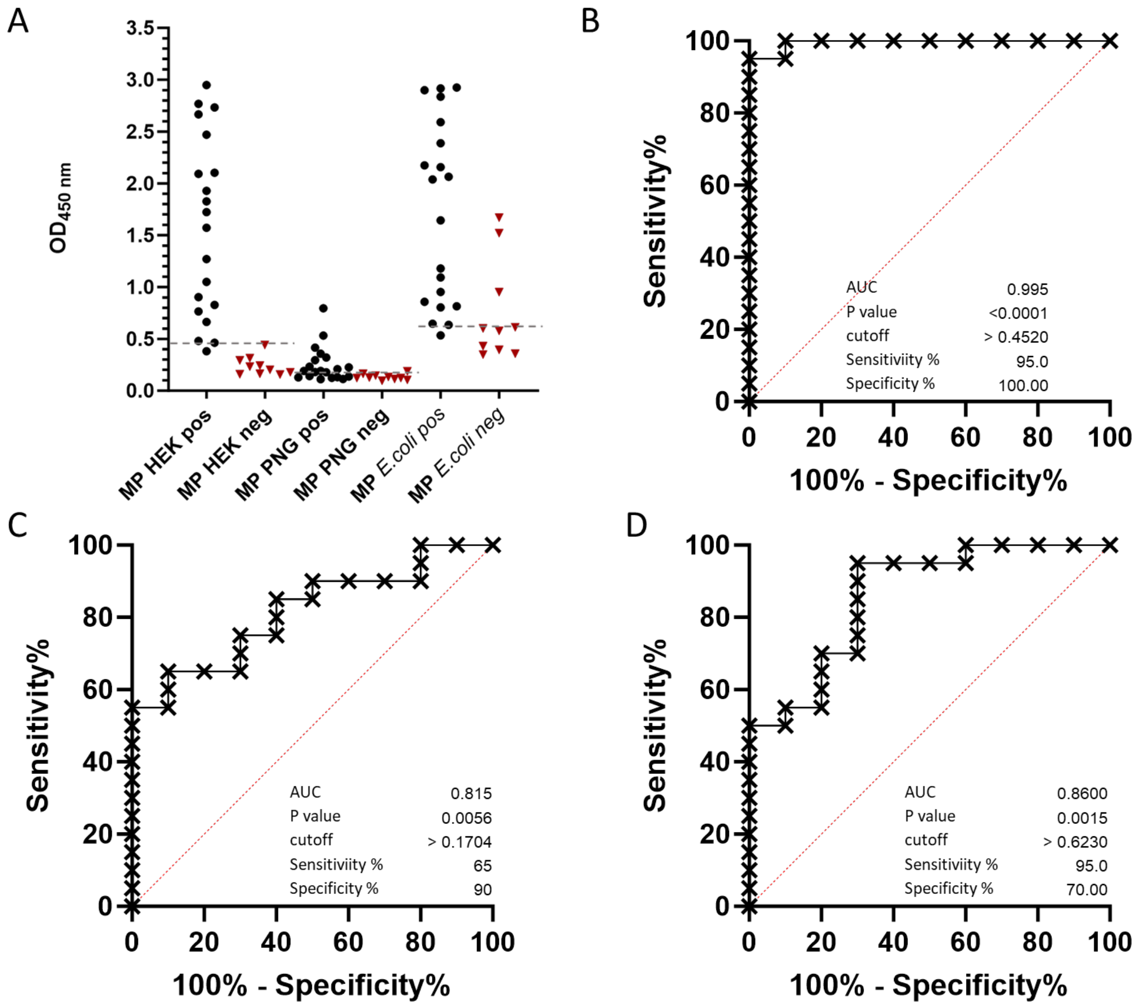

Figure S7. OD_{450} values of twenty positive sera (black) and ten negative sera (dark red) used in the IgG ELISA of the M-protein (MP) present in HEK293S lysates without (HEK) and after deglycosylation with PNGase F (PNG) and an *E. coli* lysate (A). The cutoffs are marked with a gray dashed line for the HEK M-proteins. Calculated ROC-curves (GraphPad Prism 10.0.3) of glycosylated HEK (B), deglycosylated PNG (C), and *E. coli* (D) M-proteins used in an IgG ELISA. The cutoff was determined using the Youden index.
